# The role of gasdermin-mediated mitochondrial RNA release in amplifying secondary immune response during microbial infection

**DOI:** 10.3389/fimmu.2025.1668763

**Published:** 2026-01-02

**Authors:** Uzair Afaq, Muhammad Suhaib Qudus, Siyu Liu, Kailang Wu, Yu Chen, Mingfu Tian, Jianguo Wu

**Affiliations:** 1 State Key Laboratory of Virology, College of Life Sciences, Wuhan University, Wuhan, China; 2 Key Laboratory of Ministry of Education for Viral Pathogenesis & Infection Prevention and Control, Institute of Medical Microbiology, Jinan University, Guangzhou, China; 3 Foshan Institute of Medical Microbiology, Foshan, China

**Keywords:** GSDMD cleavage, mitochondrial dysfunction, mtRNA release, immune response, VISA pathway mediated secondary inflammatory response

## Abstract

Cytoplasmic RNA serves as a typical damage-associated molecular pattern (DAMP) signal; yet the mechanisms governing its release and role in inflammatory tissue damage remain poorly understood. In our study, we demonstrated that mimicking bacterial infection by lipopolysaccharide (LPS) combined with Nigericin (Ng) effectively activates Gasdermin D (GSDMD). Conversely, Vesicular Stomatitis Virus (VSV) selectively activates Gasdermin E (GSDME). Both GSDMD and GSDME form pores in the mitochondrial membrane, facilitating the release of mitochondrial RNA (mtRNA) into the cytosol. This released mtRNA is recognized by the RNA sensor Viral Interferon Stimulated Gene Activator (VISA), which subsequently induces a robust secondary inflammatory response. Importantly, the inhibition of GSDMD and GSDME prevents mitochondrial dysfunction and mtRNA release, thereby attenuating secondary inflammatory response mediated by the VISA pathway. Utilizing an experimental mice model, we found that LPS-induced lung tissue inflammation was restored by VISA knockout (VISA^-/-^) mice. Our findings highlight the potential targeting of GSDMD, GSDME, or VISA pathway signaling as a therapeutic strategy to modulate mtRNA-mediated inflammatory responses in microbial infectious diseases.

## 1 Introduction

Pathogen entry triggers complex interactions between host sensors and pathogen-derived molecules aimed at activating a strong immune response through the active networks of innate immune cells and their signaling molecules (1, 2). Innate immune cells, comprising neutrophils, dendritic cells, and macrophages, express pattern-recognition receptors (PRRs)—evolutionarily conserved, germ-line-encoded sensors that recognize DAMPs or pathogen-associated molecular patterns (PAMPs). PRRs, such as C-type lectin receptors (CLRs), RIG-I-like receptors (RLRs), nucleotide-binding oligomerization domain (NOD)-like receptors (NLRs), Toll-like receptors (TLRs), and DNA-sensing molecules, recognize PAMPs in different subcellular compartments, including the endocytic vesicles, cell surface, and cytoplasm (3–6). Most TLRs and NLRs are critical for detecting bacterial pathogens, while DNA sensors and RLRs are mainly responsible for recognizing viral infections (7–10).

PRRs, including NLRP1, NLRP3, AIM2 (Absent in Melanoma 2), and pyrin, recruit the ASC (apoptosis-associated speck-like protein containing a caspase-recruitment domain) bipartite adaptor protein, which facilitates the assembly of the inflammasome complex. This assembly triggers caspase-1 activation through proximity-induced autocatalytic cleavage. The activated caspase-1 then processes pro-inflammatory cytokines, comprising pro-IL-18 and pro-IL-1β, converting them into their mature, biologically active forms (9, 11–13). Active caspase-1 also activates or cleaves GSDMD, releasing its N-terminal domain, which inserts into the plasma membrane, forming pores that lead to pyroptotic cell death (14–17).

RNA viruses generate replicative intermediates during replication, which are detected by the double-stranded RNA-dependent protein kinase (PKR) (18). PKR activation subsequently triggers caspase-8 and caspase-3, inducing the cleavage of GSDME and the formation of pores in the cell membrane while also promoting the inactivation of GSDMD (19). GSDMD and GSDME N-terminal fragments also translocate to the mitochondria, localizing to the mitochondrial membrane and inducing pore formation. Mitochondrial damage increases the reactive oxygen species (ROS) production, impairment of membrane potential, and mitochondrial DNA release (20–23).

Cyclic GMP-AMP synthase (cGAS), which is a sensor of DNA, recognizes self- and non-self-DNA to initiate cell damage. cGAS, after binding with cytoplasmic double-stranded DNA, catalytically converts adenosine 5’-triphosphate (ATP) and guanosine 5’-triphosphate (GTP) into cyclic GMP-AMP (cGAMP) (24, 25). The secondary messenger cGAMP binds to the stimulator of interferon genes (STING), thereby initiating a critical inflammatory response via the induction of type I interferon (IFNs) production (24, 26). The cytosolic release of mtDNA, facilitated by GSDMD and GSDME N-terminal-mediated mitochondrial membrane pore formation, stimulates the cGAS-STING pathway (21, 23).

Mitochondria, whose ancestors are considered endosymbiotic bacteria, conserve innate prokaryotic features, including a circular genome. Mammalian mitochondrial DNA is, therefore, transcribed bidirectionally, generating overlapping transcripts that can form extended double-stranded RNA structures (27, 28). However, during infectious diseases, the mechanism of mtRNA release into the cytoplasm, where self-mtRNA is recognized as DAMP, and activation of the inflammatory response remains unclear.

In this investigation, we found the role of GSDMD and GSDME in response to lipopolysaccharide (LPS) + Nigericin (Ng) treatment and vesicular stomatitis virus (VSV) infection. Our study confirms that the cleavage of GSDMD and GSDME into their N-terminal fragments localizes to mitochondria, where it leads to mitochondrial membrane pore formation and release of mtRNA into the cytosol, which activates the VISA (MAVS) pathway in the cytoplasm and produces interferon-β (IFN-β) and interleukin-6 (IL-6) and leads to lung inflammation. These findings underscore the importance of GSDMD and GSDME in regulating mitochondrial function and secondary inflammatory response via mtRNA release and activation of the VISA pathway.

## 2 Materials and methods

### 2.1 *In vivo* LPS injection

The C57BL/6 WT mice used in this study were acquired from the Hubei Research Center of Laboratory Animal (Wuhan, China), and Dr. Hongbin Shu from Wuhan University generously provided VISA^-/-^ C57BL/6 mice. All experiments involving mice were carried out with the consent of the Animal Care and Use Committee of Wuhan University (IACUC: protocol number WP20210527) and performed at the ABSL-3 laboratory of Wuhan University. Mice aged 6 to 8 weeks were assigned at random for peritoneal LPS (10mg/Kg) injection. After 12 hours, the mice were anaesthetized by Intraperitoneal injection of tribromoethanol (0.2 mL per 10 g of body weight; Beijing Jitian Biotechnology Co., Ltd., Cat No. JT0781), followed by a duration of a few minutes to allow for distribution, then euthanized by cervical dislocation upon confirmation of deep anesthesia (absence of pedal reflex). Lung tissue was harvested for further analysis.

### 2.2 Isolation of mice peritoneal macrophage cells

Fluid Thioglycolate Medium (Millipore Sigma, Product No. 1.08191.0500) was prepared as a 6% solution in double-distilled water (ddH_2_O). The solution was autoclaved to ensure sterility. One milliliter of the sterile medium was injected into the peritoneal cavity of C57BL/6 WT and C57BL/6 WT VISA^-/-^ mice. After three days, peritoneal macrophages were harvested by lavage with 1X sterile PBS and cultured in Dulbecco’s Modified Eagle’s Medium (DMEM) (Gibco) medium for further experiments.

### 2.3 Cell culture

THP-1 (human acute monocytic leukemia cells) and A549 (human lung adenocarcinoma cells) cell lines were acquired from the ATCC (American Type Culture Collection). They were cultured in Roswell Park Memorial Institute 1640 (RPMI 1640) medium (Gibco). A549 cells were cultured in Dulbecco’s Modified Eagle’s Medium (DMEM) from Gibco. Both media were enriched with 100 U/mL penicillin, 100 μg/mL streptomycin sulfate, and 10% fetal bovine serum (FBS) (Gibco). Cells were incubated in 5% CO_2_ at 37 °C in a humidified atmosphere to ensure optimal growth conditions.

### 2.4 Reagents and antibody sources

The antibodies used in this study were against GAPDH (G9295) monoclonal mouse antibody at a dilution of 1:5000, sourced from Sigma. monoclonal rabbit antibody against Tom-20 (A19403) at a dilution of 1:2000, sourced from ABclonal Technology. Monoclonal rabbit antibodies against GSDME (ab215191) were purchased from Abcam at a dilution of 1:2000, and monoclonal rabbit antibodies against GSDMD (97558) from Cell Signaling Technology at a dilution of 1:1000, monoclonal rabbit antibodies against phospho-TBK1 (ab109272) from Abcam at a dilution of 1:2000, monoclonal rabbit antibody against Caspase-1 (3866) purchased from Cell Signaling Technology at a dilution of 1:1000, monoclonal rabbit antibody against NLRP3 (15101) sourced from Cell Signaling Technology at a dilution of 1:1000 and monoclonal antibody against VDAC1 (55259-1-AP) from Proteintech.

Reagents used included IMT1 (T8841) from Targetmol, diluted in DMSO at a concentration of 10 mM. DMF (T0492) sourced from Targetmol at a concentration of 50 mM was also diluted in DMSO. LPS (L2630) from Sigma-Aldrich was prepared in ddH_2_O at 100 μg/mL. Nigericin (tlrl-nig) from Invivogen was diluted in ethanol at a concentration of 5 mM. Poly(dA-dT) (tlrl-patn) from Invivogen, diluted in ddH_2_O, at 1mg/mL concentration. VX-765 (inh-vx765i-1) from Invivogen, diluted in DMSO at a concentration of 10 mM. PMA (Phorbol 12-myristate 13-acetate) (TQ0198) from Targetmol, diluted in DMSO at a 100 μg/mL concentration. THP1 monocyte cells were treated with PMA at 100 ng/mL and incubated for 24 hours for differentiation into macrophages. Digitonin (ST1272) from Beyotime, dilute in ddH_2_O 5% (w/v).

### 2.5 Virus infection and sample collection

The samples were harvested and deactivated after viral infection, along with their supernatant, and were kept for 45 minutes at 56°C. Samples intended for western blotting were processed with lysis buffer and SDS loading buffer, while RNA samples were processed using Trizol reagent. Both heated for 15 minutes at 100°C for inactivation, samples were sent to a BSL-2 laboratory at Hubei Center for Disease Control (Wuhan, China), for further examination. Vesicular Stomatitis Virus (VSV) strain with green fluorescent protein (GFP) expression was generously provided by a researcher Dr. Bo Zhong affiliated with Wuhan University.

### 2.6 Isolation of cytosolic RNA by digitonin

THP1 and mice PM cells treated with LPS + Ng. A549, and mice PM cells infected with VSV, after incubation and collection, cells were washed two times using 1X PBS and suspended again in a cold Digitonin buffer (150 mM NaCl, 50 mM HEPES pH 7.4) including phosphatase and protease inhibitors, 1μL of Digitonin reagent was added per 1mL of Digitonin buffer to achieve specifically permeabilization of the plasma membrane without disturbing other membrane-enclosed organelles. For 20 minutes, the suspension was kept on ice and mixed gently to release cytoplasmic contents selectively. The samples underwent centrifugation at 4°C for 5 minutes at 800 rpm, the isolated supernatant (cytoplasmic fraction), and Trizol reagent were added for further RNA extraction according to the manufacturer’s protocol.

### 2.7 Western blotting

The cells were mixed into a lysis solution that comprised 150 mM NaCl, 0.5 mM EDTA, 50 mM Tris-HCl (pH 7.5), 1% SDS, and 1% NP40 for extraction and purification of proteins from the cells, furthermore, a 1:100 dilution of a protease and phosphatase inhibitor cocktail was added, sourced from Roche, followed by stirring the cells lysed mixture for 1 hour at 4°C, then supernatant was separated and heated for 5 minutes in a protein loading buffer, then put through SDS PAGE. Cells were collected and treated with a Mitochondrial Isolation Kit for Mammalian Cells (89874) from Thermo Scientific to isolate mitochondrial and cytoplasmic fractions, according to the protocol provided by the company. The cytoplasmic fraction was isolated from the supernatant, while the mitochondrial fraction was collected as a pellet; mitochondrial pellets were treated with NP40 with 1:100, a protease and phosphatase inhibitor cocktail, and heated for 5 minutes in a protein loading buffer, then put through SDS PAGE, after being transferred to PVDF (Polyvinylidene Fluoride) membrane and submerged in a 5% milk blocking solution in TBST (Tris-buffered saline with Tween 20) for 1 hour, then washed two times with TBST and added primary antibody diluted 1X PBS and 5% BSA (bovine serum albumin) at 4 °C overnight.

Membranes were rinsed twice with TBST, each time after 10 minutes of incubation, after being exposed to secondary antibodies linked to HRP for 1 hour, and rinsed again with TBST. Finally, the membranes were visualized using a LAS-4000 Imager or an X-ray film.

### 2.8 Reverse transcription and quantitative PCR: analysis and primer information

Isolation of total RNA from designated cells was performed using Trizol reagent (Invitrogen Life Technologies, Carlsbad, CA, USA) following the manufacturer’s recommended protocol. Cytoplasmic RNA was isolated using the digitonin method. For complementary DNA (cDNA) synthesis, one µg of RNA was used with HiScript II qRT Supermix (Vazyme Biotech Co., Nanjing, China). Preparation of the PCR reaction mixture involved the following components: 8 µL of RNase-free water, 10 µL of SYBR Green PCR master mix, 1 µL of a 10 µM primer solution, and 1 µL of the synthesized cDNA, by using comparative 2−ΔΔCT technique the gene expression was determined, using GAPDH as a reference gene for mRNA quantification. The following conditions were used to perform the qPCR analysis: 40 cycles, 5 minutes at 42°C, 10 seconds at 95°C, and 5 seconds at 95°C, and a Roche LC480 instrument was used. The qPCR primers used in this study are presented in **Table 1**.

**Table 1:** The primers used in this study.

Human GAPDH Forward:	Human GAPDH Reverse:
5′-GGAGCGAGATCCCTCCAAAAT-3′	5′-GGCTGTTGTCATACTTCTCATGG-3′
Human IFN-β Forward:	Human IFN-β Reverse:
5′-ATGACCAACAAGTGTCTCCTCC-3′	5′-GGAATCCAAGCAAGTTGTAGCTC-3′
Human IL-6 Forward:	Human IL-6 Reverse:
5′-ACTCACCTCTTCAGAACGAATTG-3′	5′-CCATCTTTGGAAGGTTCAGGTTG-3′
Human ND5 Forward:	Human ND5 Reverse:
5′-TCGAAACCGCAAACATATCA-3′	5′-CAGGCGTTTAATGGGGTTTA-3′
Human ND6 Forward	Human ND6 Reverse
5′-CCAATAGGATCCTCCCGAAT-3′	5′-AGGTAGGATTGGTGCTGTGG-3′
Human CYTB Forward	Human CYTB Reverse
5′-AGACAGTCCCACCCTCACAC-3′	5′-GGTGATTCCTAGGGGGTTGT-3′
Mouse GAPDH Forward:	Mouse GAPDH Reverse:
5′-AGGTCGGTGTGAACGGATTTG-3′	5′-GGGGTCGTTGATGGCAACA-3′
Mouse IFN-β Forward:	Mouse IFN-β Reverse:
5′-AGATCAACCTCACCTACAGG-3′	5′-TCAGAAACACTGTCTGCTGG-3′
Mouse IL-6 Forward:	Mouse IL-6 Reverse:
5′-TTCCATCCAGTTGCCTTCTTG-3′	5′-AATTAAGCCTCCGACTTGTGAA-3′
Mouse ND1 Forward	Mouse ND1 Reverse
5′-GAAGCGTGGATAAGATGCTC-3′	5′-CGCCCTAACAACTATTATCTTCC-3′
Mouse COX1 Forward	Mouse COX1 Reverse
5′-CCTTTGCTTCAAAACGAGAA-3′	5′-ATAGGTTGGTTCCTCGAATG-3′
Mouse CYTB Forward	Mouse CYTB Reverse
5′-GTACTGAATCCTAGTAGCCAA-3′	5′-AGTATGAGATGGAGGCTAGT-3′

### 2.9 Chemical treatment, infection, and transfection of cell lines

THP1 and PM cells were pretreated with 20 μM VX-765 for 3 hours for caspase-1 inhibition, primed with 100 ng/mL LPS for 2 hours, and treated with 5 μM Ng for one hour. For GSDMD inhibition, THP1 and PM cells were first primed with LPS, then treated with 100 μM DMF for 2 hours, and then treated with Ng. Cells primed with LPS were then transfected with 2 μg Poly(dA-dT) using Lipofectamine 2000 (Invitrogen) and incubated for 4 hours.

A549 cells were pre-treated with 100 μM DMF for 4 hours, and PM cells were pretreated with 100 μM DMF for 2 hours and then infected with VSV (VSV: 20 μL, MOI = 1) for 12 hours. Cells treated with LPS + Ng or infected with VSV had their cytoplasmic RNA extracted, then transfected into fresh cells with 2 to 4 μg using Lipofectamine and incubated for 12 hours.

### 2.10 Fluorescence microscopy

After the designated treatment and incubation, cells were washed twice with 1X PBS and stained with the JC-1 dye (Beyotime, C2006) according to the manufacturer’s protocol.

### 2.11 Flow cytometry

Mitochondrial membrane potential was detected using the JC-1 dye (Beyotime, C2006). The staining was performed following the manufacturer’s directions. Flow cytometry assessed the cells (Beckman Coulter, Cytoflex LX, USA). Flow cytometry was conducted in three replicates, and at least 10,000 cells were counted.

### 2.12 Figures and Graphic Preparation

Figures were arranged and prepared using Adobe Illustrator 2025. The graphical presentation of the experiment is displayed in **Figure 4**, **supplementary Figure S4**, and the Graphical Summary in **Figure 6** was created using BioRender (https://biorender.com).

### 2.13 Statistical analysis

Statistical analyses were conducted using Prism 8.3 (GraphPad Software Inc.). All data are presented as means ± standard deviation (SD) to represent group variability. Unpaired t-tests were applied to the data to compare two groups, assuming a normal distribution. The criteria for statistical significance were established as follows: *p < 0.05, **p < 0.01, ***p < 0.001 and ****p < 0.0001, indicating progressively higher levels of significance. Non-significant data is indicated as ns (p > 0.05).

## 3 Results

### 3.1 Effects of GSDMD and GSDME inhibition on cytokine production

**Figure 1:**
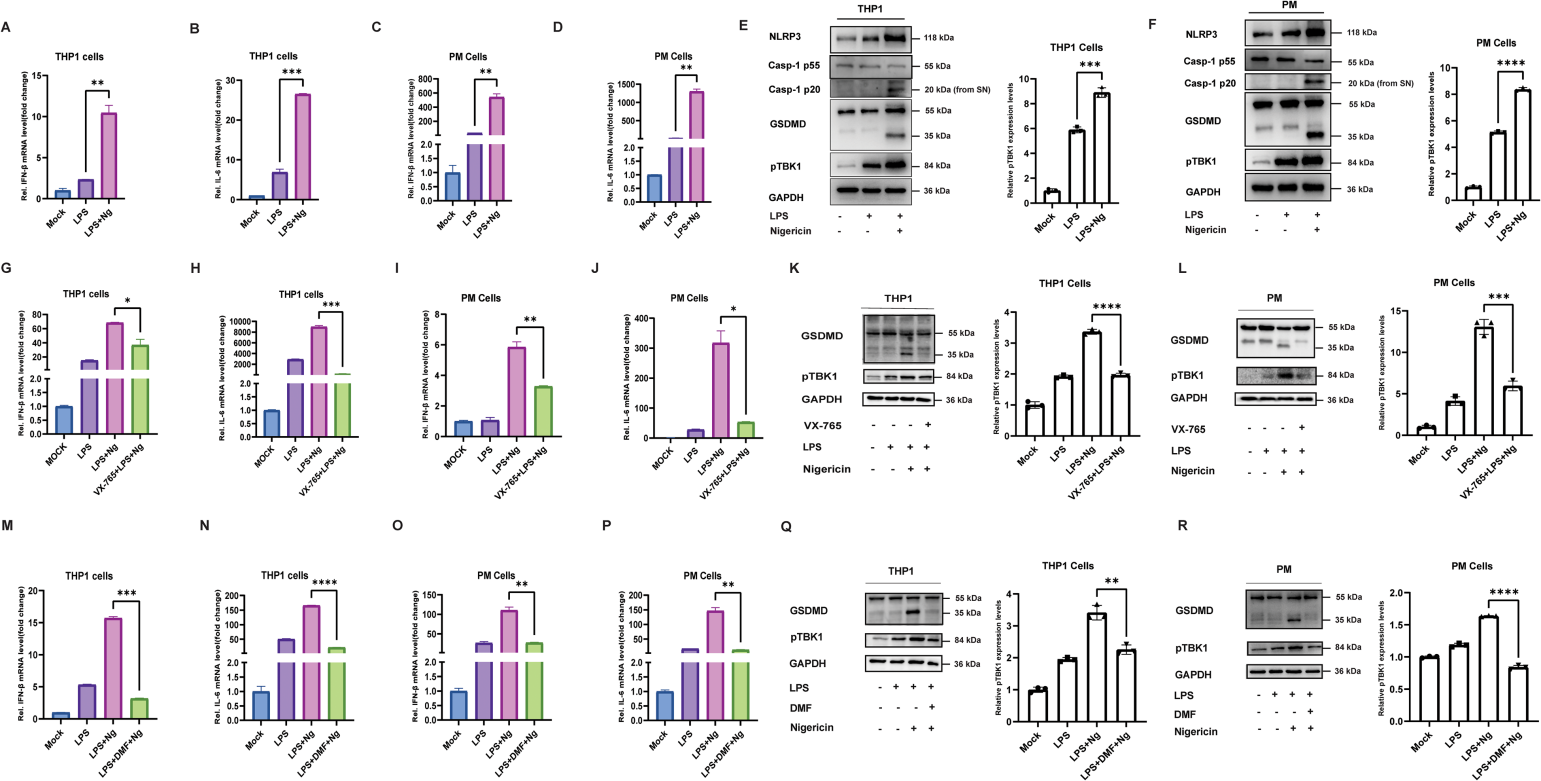
GSDMD inhibition modulates cytokine production in LPS and Ng responses. **(A, B)** THP1 cells were primed with 100 ng/mL LPS for 2 hours and activated with 5 μM/mL nigericin (Ng) for one hour, with qPCR analysis demonstrating elevated expression of IFN-β **(A)** and IL-6 **(B)**. An unpaired t-test determined statistical significance. Data represent ± SEM (**p < 0.01, ***p < 0.001). **(C, D)** mouse peritoneal macrophages (PM cells) primed with 100 ng/mL LPS for 2 hours, treated with 5 μM/mL Ng for one hour, qPCR analysis demonstrating elevated expression of IFN-β **(C)** and IL-6 **(D)**, an unpaired t-test determined statistical significance. Data represent ± SEM (**p < 0.01, **p < 0.01). **(E, F)** THP-1 and PM cells primed with 100 ng/mL LPS alone exhibited reduced NLRP3 activation, caspase-1, and GSDMD cleavage. In contrast, cells primed with LPS and activated with 5 μM Ng showed enhanced NLRP3 activation, caspase-1, and GSDMD cleavage, leading to TBK1 phosphorylation. Cleaved caspase-1 protein (p20) was isolated from the supernatant (SN), and pTBK1 protein expression levels were quantified using ImageJ; an unpaired t-test determined statistical significance. Data represent ± SEM (***p < 0.001, ****p < 0.0001). **(G, H)** THP1 cells primed with 100 ng/mL LPS alone, cells treated with LPS + Ng, cells pretreated with 20 μM VX-765 for 2 hours and then treated with LPS + Ng, qPCR analysis showing decreased IFN-β **(E)** and IL-6 **(F)** expression in VX-765 pretreated THP-1 cells. Data represent mean ± SEM (*p < 0.05, ***p < 0.001). **(I, J)** PM cells primed with LPS alone, cells treated with LPS + Ng, cells pretreated with 20 μM VX-765 for 2 hours and then treated with LPS + Ng, qPCR analysis showing decreased IFN-β **(G)** and IL-6 **(H)** expression in VX-765 pretreated PM cells. Data represent mean ± SEM (**p < 0.01, *p < 0.05). **(K, L)** Western blot analysis of THP1 and PM cells showed no GSDMD cleavage and TBK1 phosphorylation in LPS-primed cells alone and cells pretreated with 20 μM VX-765 for 2 hours followed by LPS + Ng treatment. In contrast, LPS + Ng treatment induced GSDMD cleavage and elevated TBK1 phosphorylation, pTBK1 protein expression levels were quantified using ImageJ; an unpaired t-test determined statistical significance. Data represent ± SEM (****p < 0.0001, ***p < 0.001). **(M, N)** THP1 cells primed with LPS alone, cells primed with LPS, then treated with Ng, cells primed with LPS, then treated with 100 μM/mL dimethyl fumarate (DMF) for 2 hours and then treated with Ng, qPCR analysis showing decreased IFN-β **(K)** and IL-6 **(L)** expression in DMF treated THP1 cells. Data represent mean ± SEM (***p < 0.001, ****p < 0.0001). **(O, P)** PM cells primed with LPS alone, cells primed with LPS and then treated with Ng, cells primed with LPS, then treated with 100 μM/mL DMF for 2 hours and then treated with Ng, qPCR analysis showing decreased IFN-β **(M)** and IL-6 **(N)** expression in DMF treated PM cells. Data represent mean ± SEM (**p < 0.01, **p < 0.01). **(Q, R)** Western blot analysis showed that LPS priming, followed by Ng treatment, induced GSDMD cleavage and elevated TBK1 phosphorylation in THP-1 and PM cells. However, cells primed with LPS, treated with DMF, and treated with Ng inhibited GSDMD cleavage and reduced TBK1 phosphorylation, pTBK1 protein expression levels were quantified using ImageJ; an unpaired t-test determined statistical significance. Data represent ± SEM (****p < 0.0001, ***p < 0.001), all experiments were repeated three times, and representative experiments are shown.

The innate immune response is activated by recognizing PAMPs and DAMPs, which activate key signaling pathways, inducing inflammasome activation and type I interferon response. These pathways are essential for producing pro-inflammatory cytokines, such as IFN-β and IL-6, which mediate the immune response to cellular stress and microbial infection (29, 30). In addition, cells treated with LPS and Ng trigger the activation of the NLRP3 pathway, leading to the cleavage of caspase-1, which in turn cleaves GSDMD (31). Conversely, cells infected with VSV activate the PKR pathway and caspase-3, resulting in the cleavage of GSDME (19, 32). Our study observed that THP1 cells primed with LPS and then activated with Ng significantly induced the production of IFN-β and IL-6 (**Figures 1A, B**). A similar increase in IFN-β and IL-6 expression was observed in mouse peritoneal macrophages (PM cells) after treatment with LPS + Ng (**Figures 1C, D**), indicating the activation of the NLRP3 inflammasome and type I interferon pathways. To further confirm that the activation of NLRP3, cleavage of caspase-1, and GSDMD leads to phosphorylation of TANK-binding kinase 1 (TBK1), Western blot analysis of THP-1 and PM cells revealed that LPS priming alone did not induce activation of NLRP3, cleavage of caspase-1 and GSDMD, or elevate TBK1 phosphorylation, suggesting minimal inflammasome activation. In contrast, THP-1 and PM cells primed with LPS and subsequently activated with Ng exhibited NLRP3 activation, cleavage of caspase-1 and GSDMD, and increased levels of TBK1 phosphorylation, demonstrating that treatment with Ng following LPS priming causes the cleavage of caspase-1 and GSDMD, leading to downstream type I interferon production (**Figures 1E, F**). A549 cells infected with VSV were also observed to significantly increase IFN-β and IL-6 expression (**Supplementary Figures S1A, B**). A similar response was observed in PM cells infected with VSV (**Supplementary Figures S1C, D**), confirming the consistent activation of the same type I interferon pathway across different cell types and treatments. Caspase-1 plays an essential role in inflammasome activation, as revealed by further investigation. THP1 cells pretreated with VX-765 (a caspase-1 inhibitor), primed with LPS, and then treated with Ng, compared to THP1 cells treated with LPS + Ng alone, showed that the cells pretreated with VX-765 significantly reduced the production of IFN-β and IL-6 (**Figures 1G, H**). A similar experiment performed in PM cells pretreated with VX-765 also showed a similar reduction in cytokine expression (**Figures 1I, J**). This indicates that caspase-1 is essential for the expression of IFN-β and IL-6. Our study further confirmed the activation of caspase-1 by using poly(deoxyadenylic-deoxythymidylic) acid (poly(dA-dT)). In both THP-1 and PM cells, VX-765 pretreatment reduced IFN-β and IL-6 expression following LPS priming and poly(dA-dT) treatment, compared to THP-1 and PM cells treated with LPS + poly(dA-dT) only, confirming the critical role of caspase-1 activation in cytokine production (**Supplementary Figures S1E, SF, S1G, S1H**). Western blot analysis of THP-1 cells revealed that LPS priming alone did not induce GSDMD cleavage or elevate TANK-binding kinase 1 (TBK1) phosphorylation, suggesting minimal inflammasome activation. THP1 cells pretreated with VX-765 showed no GSDMD cleavage and reduced levels of TBK1 phosphorylation. In contrast, THP1 cells treated with LPS + Ng alone induced GSDMD cleavage and increased TBK1 phosphorylation, demonstrating that cells pretreated with VX-765 blocks GSDMD activation and downstream type I interferon production (**Figure 1K**). Similar experiments were performed in PM cells, showing the same results for the western blot analysis, confirming that TBK1 phosphorylation depends on the cleavage of GSDMD (**Figure 1L**). The succination of GSDMD and GSDME by dimethyl fumarate (DMF) leads to alterations in their structural conformation, which results in the inhibition of caspase interaction with GSDMD and GSDME, subsequently inhibiting their cleavage (33). To further understand, we investigated the role of GSDMD and GSDME by using DMF to inhibit their cleavage. THP1 cells primed with LPS, treated with DMF, and then Ng treatment significantly reduced the IFN-β and IL-6 expression compared to THP1 cells treated with LPS + Ng only (**Figures 1M, N**). After being primed with LPS, PM cells were treated with DMF and then exposed to Ng showed similar results (**Figures 1O, P**). These results confirm that the expression of IFN-β and IL-6 depends on GSDMD cleavage. A549 cells pretreated with DMF and followed by VSV infection showed significantly reduced expression of IFN-β and IL-6 compared to A549 cells infected with VSV alone (**Supplementary Figures S1I, J**). Similar results were found in the PM cells pretreated with DMF, followed by VSV infection, compared to those infected with VSV alone (**Supplementary Figures S1K, L**). These results confirm in A549 and PM cells that the expression of IFN-β and IL-6 depends on GSDME cleavage. Western blot analysis of THP-1 cells showed that LPS priming followed by DMF treatment and Ng exposure significantly inhibited GSDMD cleavage and reduced TBK1 phosphorylation, compared to cells treated with LPS + Ng alone, where efficient GSDMD cleavage and elevated TBK1 phosphorylation were observed (**Figure 1Q**). A similar trend was observed in PM cells, confirming that GSDMD cleavage is crucial for TBK1 phosphorylation and activation (**Figure 1R**). In A549 cells, VSV infection induced GSDME cleavage and elevated TBK1 phosphorylation. However, DMF pretreatment significantly inhibited both GSDME cleavage and TBK1 phosphorylation, highlighting the role of GSDME cleavage in TBK1 activation during viral infection (**Supplementary Figure S1M**). Consistent results were observed in mouse PM cells (**Supplementary Figure S1N**). Collectively, our finding demonstrates that the cleavage of GSDMD and GSDME plays a pivotal role in the regulation of pro-inflammatory cytokines production, specifically IFN- β and IL-6, following innate immune activation. The inhibition of caspase-1 through VX-765, as well as the succination of GSDMD and GSDME by dimethyl fumarate (DMF), significantly reduced cytokine expression, underscoring the importance of these pathways in the immune response. Furthermore, the phosphorylation of TBK1 following the modulation of GSDMD and GSDME cleavage further confirm their involvement in the type 1 interferon singaling pathway. Thus, our study concludes that GSDMD and GSDME cleavage is essential for the effective production of pro-inflammatory cytokines in response to innate immune stimuli.

### 3.2 Regulation of mitochondrial RNA release by GSDMD and GSDME

**Figure 2:**
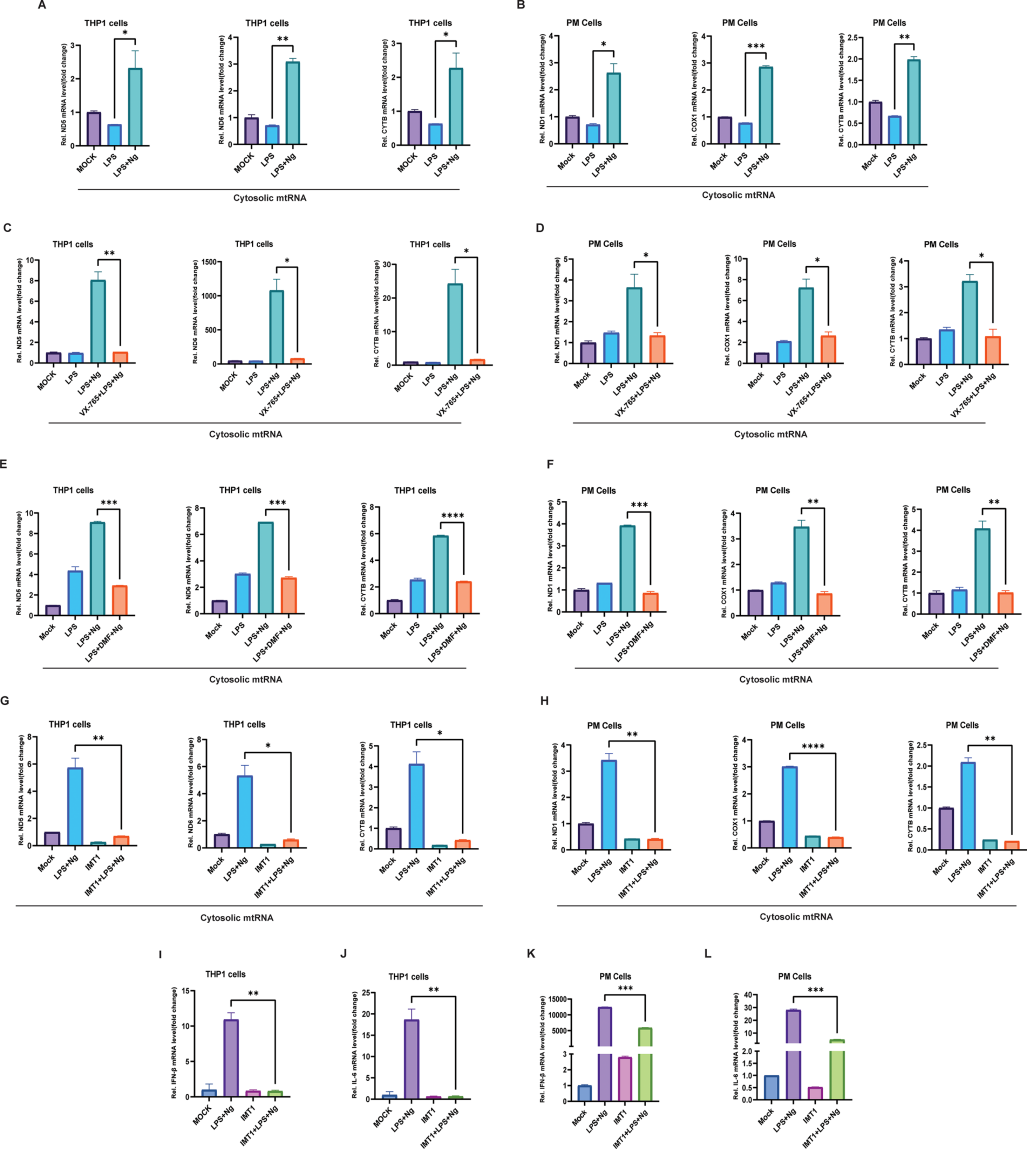
Gasdermin D cleavage leads to mitochondrial RNA release. **(A)** THP1 cells primed with LPS alone, cells treated with LPS + Ng, cytoplasmic RNA extracted, qPCR analysis showing increased mitochondrial RNA (mtRNA) (ND5, ND6, and CYTB) expression in LPS + Ng treated THP1 cells. Data represent mean ± SEM (*p < 0.05, **p < 0.01, *p < 0.05). **(B)** PM cells primed with LPS alone, cells treated with LPS + Ng, cytoplasmic RNA extracted, qPCR analysis showing increased mtRNA (ND1, COX1, and CYTB) expression in LPS + Ng treated PM cells. Data represent mean ± SEM (*p < 0.05, ***p < 0.001, **p < 0.01). **(C)** THP1 cells primed with LPS, cells treated with LPS + Ng, and cells pretreated with VX-765 then treated with LPS + Ng, qPCR analysis showing decreased mtRNA (ND5, ND6, and CYTB) expression in THP1 cells pretreated with VX-765. Data represent mean ± SEM (**p < 0.01, *p < 0.05, *p < 0.05). **(D)** PM cells primed with LPS, cells treated with LPS + Ng, and cells pretreated with VX-765 then treated with LPS + Ng, qPCR analysis showing decreased mtRNA (ND1, COX1, and CYTB) expression in PM cells pretreated with VX-765. Data represent mean ± SEM (*p < 0.05, *p < 0.05, *p < 0.05). **(E)** THP1 cells primed with LPS, cells treated with LPS + Ng, and cells primed with LPS, treated with DMF, then with Ng, qPCR analysis showing decreased mtRNA (ND5, ND6, and CYTB) expression in THP1 cells treated with DMF. Data represent mean ± SEM (***p < 0.001, ***p < 0.001, ****p < 0.0001). **(F)** PM cells primed with LPS, cells treated with LPS + Ng, and cells primed with LPS, treated with DMF, then with Ng, qPCR analysis showing decreased mtRNA (ND1, COX1, and CYTB) expression in PM cells treated with DMF. Data represent mean ± SEM (***p < 0.001, **p < 0.01, **p < 0.01). **(G)** THP1 cells primed with LPS, cells treated with LPS + Ng, cells treated with 10 μM/mL IMT1 (mitochondrial RNA polymerase inhibitor) only, and cells pretreated with 10 μM/mL IMT1 then treated with LPS + Ng, qPCR analysis showing decreased mtRNA (ND5, ND6, and CYTB) expression in THP1 cells pretreated with IMT1 then LPS + Ng. Data represent mean ± SEM (**p < 0.01, *p < 0.05, *p < 0.05). **(H)** PM cells primed with LPS, cells treated with LPS + Ng, cells treated with 10 μM/mL IMT1 only, and cells pretreated with IMT1 then treated with LPS + Ng, qPCR analysis showing decreased mtRNA (ND1, COX1, and CYTB) expression in PM cells pretreated with 10 μM/mL IMT1 then LPS + Ng. Data represent mean ± SEM (**p < 0.01, ****p < 0.0001, **p < 0.01). **(I, J)** THP1 cells primed with LPS, cells treated with LPS + Ng, cells treated with IMT1 only, and cells pretreated with IMT1 then treated with LPS + Ng, qPCR analysis showing decreased IFN-β **(I)** and IL-6 **(J)** expression in THP1 cells pretreated with IMT1 then LPS + Ng. Data represent mean ± SEM (**p < 0.01, **p < 0.01). **(K, L)** PM cells primed with LPS, cells treated with LPS + Ng, cells treated with IMT1 only, and cells pretreated with IMT1 then treated with LPS + Ng, qPCR analysis showing decreased IFN-β **(K)** and IL-6 **(L)** expression in PM cells pretreated with IMT1 then LPS + Ng. Data represent mean ± SEM (***p < 0.001, ***p < 0.001), all experiments were repeated three times, and representative experiments are shown.

Mitochondrial dysfunction has emerged as a key driver of innate immune activation, with mitochondrial RNA (mtRNA) released into the cytoplasm, triggering inflammasome activation and type I interferon responses (34) The qPCR analysis revealed elevated cytoplasmic mtRNA levels (ND5, ND6, and CYTB) in THP-1 cells treated with LPS + Ng, suggesting that GSDMD activation via LPS + Ng treatment induces mitochondrial membrane permeabilization. Similar results were observed in PM cells (ND1, COX1, and CYTB), highlighting a conserved mechanism of GSDMD-mediated mitochondrial damage in both cell types (**Figures 2A, B**). In A549 cells infected with VSV, elevated cytosolic mtRNA (ND5, ND6, and CYTB) indicated that GSDME cleavage by VSV infection contributes to mitochondrial membrane disruption. Consistent findings in PM cells (ND1, COX1, and CYTB) further support the role of GSDME in mitochondrial membrane permeabilization during VSV infection (**Supplementary Figures S2A, B**). Caspase-1 inhibition with pretreatment of VX-765 significantly reduced cytoplasmic mtRNA levels in both THP-1 (ND5, ND6, and CYTB) and PM cells (ND1, COX1, and CYTB), followed by treatment with LPS + Ng compared to THP1 and PM cells treated with LPS + Ng alone (**Figures 2C, D**). These findings confirm that caspase-1-mediated GSDMD activation drives mitochondrial membrane pore formation. Similarly, caspase-1 activation by poly(dA-dT) transfection elevated cytoplasmic mtRNA levels in LPS-primed THP-1 and PM cells, while VX-765 pretreatment significantly reduced mtRNA release, further reinforcing the role of caspase-1 and GSDMD in mitochondrial damage (**Supplementary Figures S2C, D**). THP-1 and PM cells primed with LPS and treated with DMF to directly inhibit GSDMD, followed by Ng exposure, significantly reduced mtRNA levels in THP-1 (ND5, ND6, and CYTB) and PM cells (ND1, COX1, and CYTB) compared to THP-1 and PM cells treated with LPS + Ng alone (**Figures 2E, F**), thus confirming GSDMD’s role in mitochondrial permeabilization. Similarly, DMF pretreatment in A549 cells and PM cells infected with VSV reduced cytosolic mtRNA levels, confirming GSDME-mediated mitochondrial pore formation during viral infection (**Supplementary Figures S2E, F**). To further explore the transcriptional control of mitochondrial RNA, we used IMT1, a specific inhibitor of mitochondrial RNA polymerase that has been shown to reduce mitochondrial RNA synthesis effectively (35). Inhibition of mitochondrial RNA polymerase with IMT1 pretreated significantly reduced mtRNA levels in both THP-1 (ND5, ND6, and CYTB) and PM cells (ND1, COX1, and CYTB) followed LPS + Ng treatment, in contrast to THP1 and PM cells treated with LPS + Ng alone (**Figures 2G, H**), demonstrating that IMT1 prevents mtRNA accumulation by blocking transcription. These results emphasize the essential role of mtRNA in driving innate immune responses. Furthermore, THP1 and PM cells pretreated with IMT1, followed by LPS + Ng treatment, significantly reduced IFN-β and IL-6, in contrast to THP1 and PM cells treated with LPS + Ng alone, thereby confirming the critical role of mtRNA in IFN-β and IL-6 expression (**Figures 2I–L**). Collectively, these observations confirm that GSDMD and GSDME cleavage induce mitochondrial membrane permeabilization and trigger a secondary inflammatory response by mtRNA release into the cytoplasm.

### 3.3 GSDMD and GSDME cleavage induces mitochondrial dysfunction

**Figure 3:**
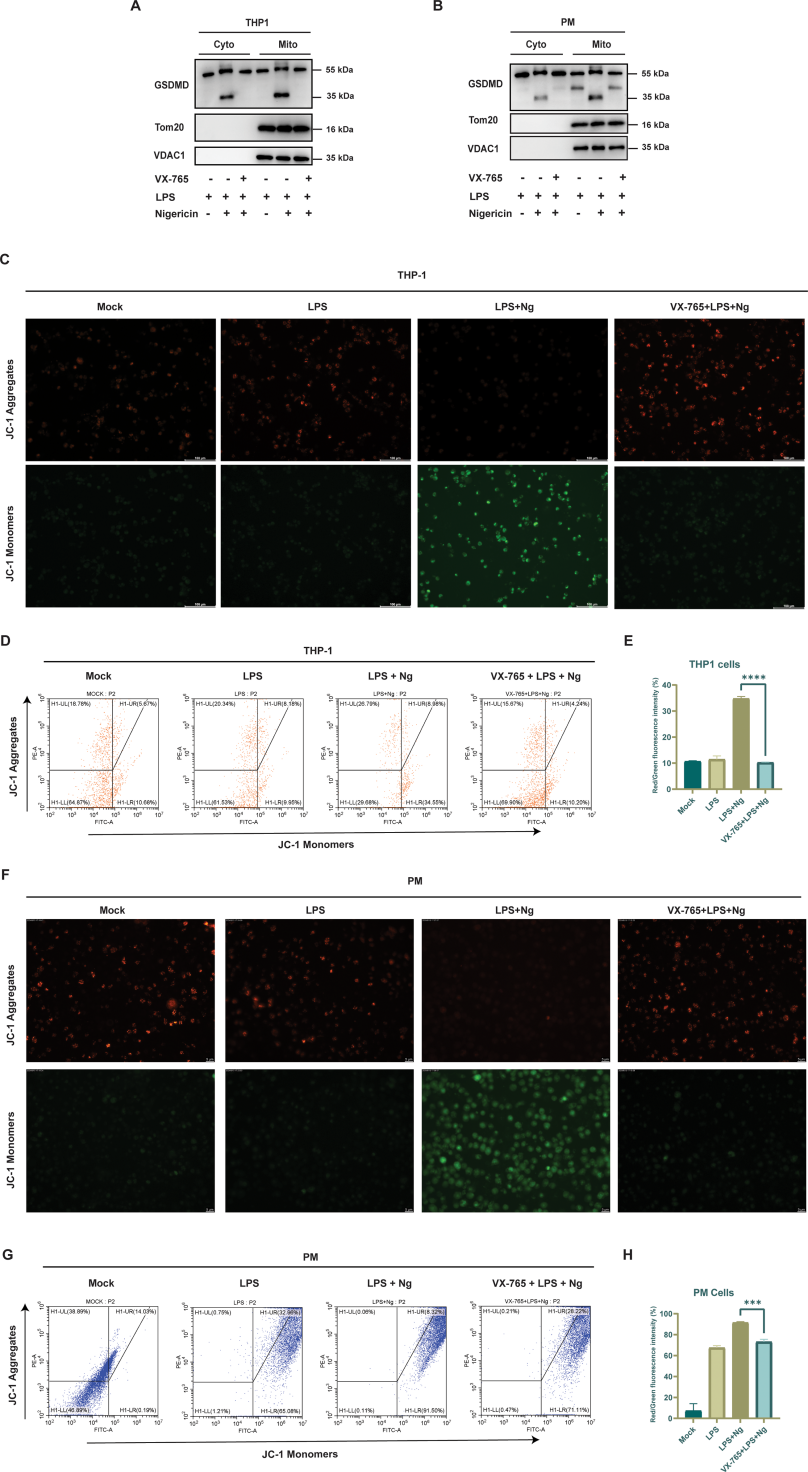
GSDMD as key drivers of mitochondrial dysfunction: **(A)** THP1 cells primed with LPS alone, cells primed with LPS, then treated with Ng, cells pretreated with VX-765, then treated with LPS + Ng. Subjected to mitochondrial Isolation, Western blot analysis of GSDMD cleavage was observed in both cytoplasmic and mitochondrial fractions of LPS + Ng-treated cells; no GSDMD cleavage was observed in cells pretreated with VX-765 or those primed with LPS alone. **(B)** PM cells primed with LPS, cells primed with LPS, then treated with Ng, cells pretreated with VX-765, then treated with LPS + Ng. Subjected to mitochondrial Isolation, Western blot analysis of GSDMD cleavage was observed in both cytoplasmic and mitochondrial fractions of LPS + Ng-treated cells; no GSDMD cleavage was observed in cells pretreated with VX-765 or those primed with LPS alone. **(C)** THP1 cells primed with LPS alone, cells primed with LPS, then treated with Ng, cells pretreated with VX-765, then treated with LPS + Ng. Cells stained with JC-1 dye, LPS + Ng alone displayed a higher proportion of JC-1 monomers and fewer aggregates, in contrast to THP-1 cells primed with LPS alone and cells pretreated with VX-765. **(D, E)** THP1 cells were primed with LPS alone, primed with LPS, then treated with Ng, pretreated with VX-765, then treated with LPS + Ng treatment, and mitochondrial damage was assessed using JC-1 staining. Flow cytometry analysis confirmed the results observed by inverted microscopy; the histogram shows the percentage. **(F)** PM cells primed with LPS alone, cells primed with LPS, then treated with Ng, cells pretreated with VX-765, then treated with LPS + Ng. Cells stained with JC-1 dye, LPS + Ng alone displayed a higher proportion of JC-1 monomers and fewer aggregates, in contrast to PM cells primed with LPS alone and cells pretreated with VX-765. **(G, H)** PM cells were primed with LPS alone, primed with LPS, then treated with Ng, pretreated with VX-765, then treated with LPS + Ng treatment, and mitochondrial damage was assessed using JC-1 staining. Flow cytometry analysis confirmed the results observed by inverted microscopy; the histogram shows the percentage, all experiments were repeated three times, and representative experiments are shown. The histogram shows the percentage of Flow Cytometry analysis, with statistical significance determined by an unpaired t-test. Data represent ± SEM (***p < 0.001, ****p < 0.0001).

Caspase-1 and caspase-3 activation are essential events that trigger GSDMD and GSDME cleavage and generate their N-terminal fragments, which translocate to mitochondria. Once localized to the mitochondria, GSDMD and GSDME N-terminal fragments induce mitochondrial damage and drive the inflammatory response (21, 22, 31). THP1 cells pretreated with VX-765, followed by LPS priming and Ng exposure, THP1 cells primed with LPS only, and THP1 cells primed with LPS and Ng exposure only, underwent cytoplasmic and mitochondrial fractions separation using the mitochondrial isolation Kit for mammalian cells (Thermo Scientific). Western blot analysis of THP1 cells treated with LPS + Ng revealed GSDMD cleavage in cytosolic and mitochondrial fractions, with GSDMD N-terminal and full-length fractions detected in mitochondrial fractions. In contrast, GSDMD cleavage was not detected in cells pretreated with VX-765 or primed with LPS alone. These findings indicate that GSDMD cleavage and its translocation to mitochondria occur specifically upon LPS + Ng treatment and depend on caspase-1 activity (**Figure 3A**). A similar trend was seen in PM cells (**Figure 3B**). To further elucidate the role of GSDME in mitochondrial damage. A549 cells were first infected with VSV alone and another set of A549 cells were pretreated with DMF following VSV infection. Western blot analysis revealed that GSDME cleavage was observed in the cytoplasmic and mitochondrial fractions of VSV-infected cells, with full-length GSDME and its N-terminal fragment detected in the mitochondrial fraction. In contrast, no GSDME cleavage was observed in DMF-pretreated cells (**Supplementary Figure S3A**). Consistent results were obtained in PM cells (**Supplementary Figure S3B**). These findings prove that the cleavage and activation of GSDMD and GSDME occur. Both gasdermins translocate to the mitochondria, where their N-terminal fragments induce pore formation in the mitochondrial membrane, destabilizing and damaging it. To assess mitochondrial damage in THP-1 cells, we performed JC-1 staining followed by inverted microscopy analysis. Cells treated with LPS + Ng alone exhibited a higher proportion of JC-1 monomers (green fluorescence) and fewer JC-1 aggregates (red fluorescence), indicative of significant mitochondrial damage and membrane depolarization. In contrast, cells primed with LPS alone and cells pretreated with the VX-765 before LPS + Ng treatment showed a reduction in JC-1 monomers and an increase in JC-1 aggregates, suggesting reduced mitochondrial dysfunction. These findings indicate that VX-765 mitigates mitochondrial damage by inhibiting caspase-1 activation, thereby preventing GSDMD-mediated mitochondrial membrane disruption (**Figure 3C**). Flow cytometry analysis of the same treated THP-1 cells further confirmed these findings, showing fewer JC-1 monomers and more aggregates in VX-765-pretreated cells compared to LPS + Ng-treated cells (**Figures 3D, E**). Similarly, inverted microscopy revealed that LPS + Ng treatment in PM cells increased JC-1 monomers and decreased aggregates, consistent with mitochondrial damage. VX-765 pretreatment before LPS priming and Ng treatment resulted in fewer monomers and more aggregates, indicating a protective effect against mitochondrial dysfunction (**Figure 3F**). Flow cytometry further validated the microscopy data, reinforcing the role of VX-765 in alleviating mitochondrial damage by inhibiting caspase-1 and GSDMD activation (**Figures 3G, H**). In A549 cells infected with VSV, JC-1 staining revealed increased monomers and reduced aggregates, indicating mitochondrial damage. However, DMF pretreatment before VSV infection reduced JC-1 monomers and increased aggregates, suggesting protection from mitochondrial damage. These results imply that DMF mitigates mitochondrial damage by inhibiting GSDME activation, which is involved in mitochondrial membrane disruption (**Supplementary Figure S3C**). Flow cytometry analysis corroborated these findings, showing fewer monomers and more aggregates in DMF-pretreated cells compared to VSV-infected cells (**Supplementary Figure S3D, E**). Consistent results were observed in PM cells by JC-1 staining and inverted microscopy. PM cells infected solely with VSV exhibited a rise in JC-1 monomers and a decline in JC-1 aggregates. PM cells pretreated with DMF and then infected with VSV show reduced monomers and increased aggregates (**Supplementary Figure S3F**). Flow cytometry of PM cells provides additional validation of the microscopic findings, underscoring the effectiveness of DMF in reducing mitochondrial damage by blocking the cleavage of GSDME (**Supplementary Figure S3G, H**). These findings confirm that DMF inhibits GSDME cleavage and prevents mitochondrial damage. Together, these observations highlight the essential role of GSDMD and GSDME in mitochondrial damage and the protective effects of VX-765 and DMF in reducing mitochondrial damage.

### 3.4 Mitochondrial RNA release activates the VISA pathway-mediated secondary inflammatory response

**Figure 4:**
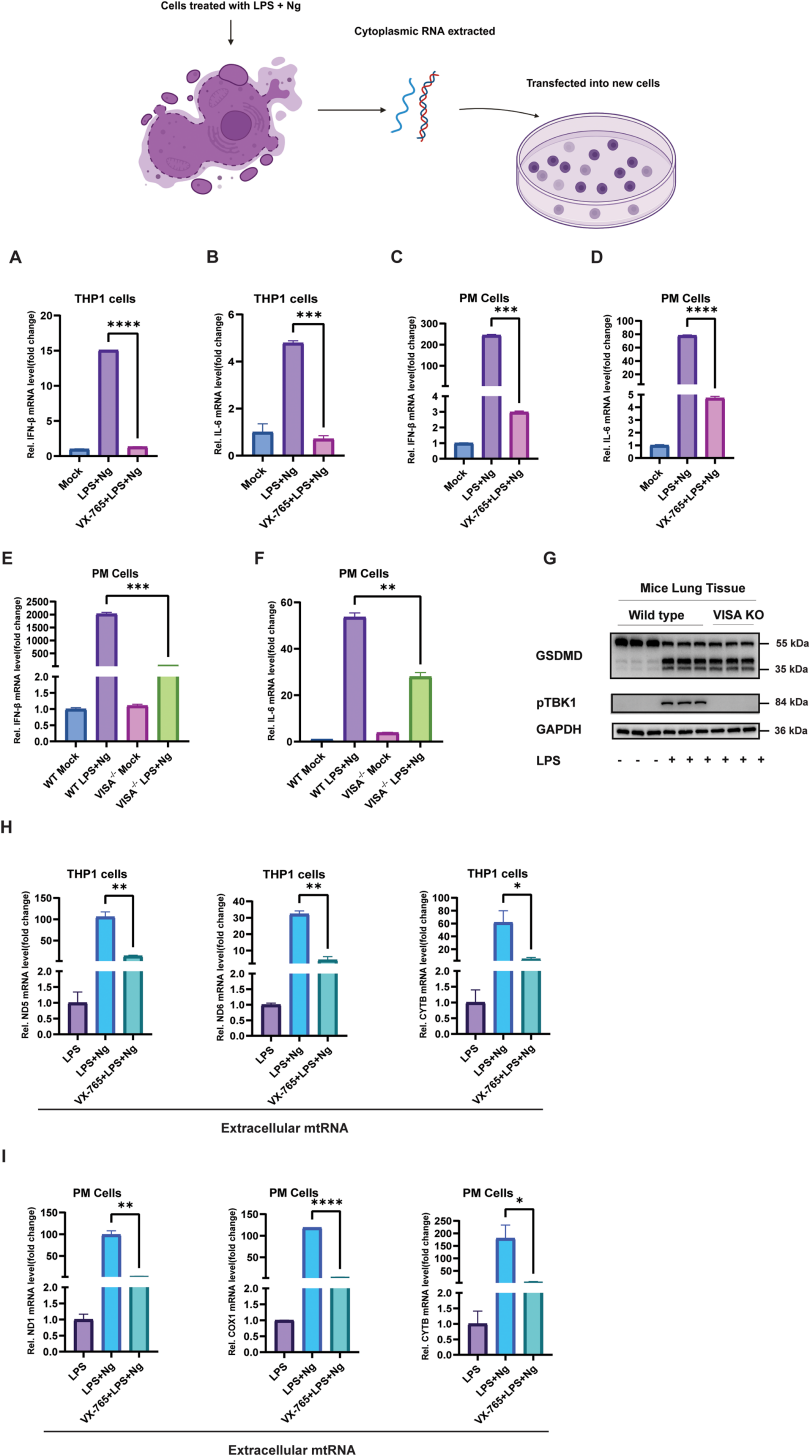
GSDMD cleavage mediates mtRNA release and secondary inflammatory responses via the VISA pathway. **(A, B)** THP-1 cells primed with LPS and treated with Ng, and cells pretreated with VX-765 and LPS + Ng had their cytoplasmic RNA extracted and 2 μg/mL transfected into fresh THP-1 cells. qPCR analysis showed significantly higher IFN-β **(A)** and IL-6 **(B)** expression in fresh THP1 cells transfected from treated with LPS + Ng alone compared to VX-765-pretreated cells. Data are presented as mean ± SEM (****p < 0.0001, ***p < 0.001). **(C, D)** PM cells primed with LPS and treated with Ng, and cells pretreated with VX-765, then treated with LPS + Ng, had their cytoplasmic RNA extracted, and 2 μg/mL transfected into fresh PM cells. qPCR analysis showed significantly higher IFN-β **(C)** and IL-6 **(D)** expression in fresh PM cells transfected from treated with LPS + Ng alone compared to VX-765-pretreated cells. Data are presented as mean ± SEM (***p < 0.001, ****p < 0.0001). **(E, F)** WT PM cells and VISA^-/-^ PM cells, primed with LPS and then treated with Ng, had their cytoplasmic RNA extracted and 2 μg/mL transfected into fresh WT PM cells and VISA^−^/^−^ PM cells. qPCR analysis showed significantly higher IFN-β **(E)** and IL-6 **(F)** expression in fresh WT PM cells than in fresh VISA^-/-^ PM cells. Data are presented as mean ± SEM (***p < 0.001, **p < 0.01). **(G)** Six WT mice (three peritoneal injected with 10 mg/kg LPS and three untreated) and three VISA^-/-^ mice peritoneal injected with 10 mg/kg LPS. Western blot analysis of lung tissues showed no GSDMD cleavage or pTBK1 expression in untreated WT mice. In contrast, LPS-injected WT mice exhibited both GSDMD cleavage and pTBK1 expression, whereas LPS-injected VISA^-/-^ mice displayed GSDMD cleavage but no pTBK1 expression. **(H)** THP-1 cells were primed with LPS alone, cells primed with LPS were then treated with Ng, and cells pretreated with VX-765, followed by LPS + Ng treatment, and their culture media was collected. RNA extraction and qPCR analysis revealed higher expression of mtRNA (ND5, ND6, and CYTB) in the extracellular space of cells treated with LPS + Ng alone compared to VX-765-pretreated cells. Data are presented as mean ± SEM (**p < 0.01, **p < 0.01, *p < 0.05). **(I)** PM cells primed with LPS only, primed with LPS, then treated with Ng, and cells pretreated with VX-765, followed by LPS + Ng treatment, had their culture media collected. RNA extraction and qPCR analysis revealed higher expression of mtRNA (ND1, COX1, and CYTB) in the extracellular space of cells treated with LPS + Ng alone compared to VX-765-pretreated cells. Data are presented as mean ± SEM (**p < 0.01, ****p < 0.0001, *p < 0.05), all experiments were repeated three times, and representative experiments are shown.

Mitochondrial RNA is pivotal in activating the VISA pathway, essential for type 1 interferon production (36). THP-1 cells primed with LPS and treated with Ng, and another set of THP-1 cells were pretreated with VX-765, primed with LPS, following Ng treatment. The cytoplasmic RNA was extracted and transfected into fresh THP1 cells following 12 hours of incubation. The qPCR analysis revealed elevated expression of IFN-β and IL-6, in cells transfected with cytoplasmic RNA from LPS + Ng treated alone (**Figures 4A, B**). Similar experiments were performed with PM cells, and consistent results were observed; cytoplasmic RNA transfection from PM cells treated with LPS + Ng alone into fresh PM cells revealed significantly higher expression of IFN-β and IL-6, in contrast to PM cells pretreated with VX-765 followed by LPS + Ng treatment and cytoplasmic RNA transfection into fresh PM cells (**Figures 4C, D**). These findings support the conclusion that cytoplasmic mtRNA released upon GSDMD activation is sufficient to propagate an inflammatory response (IFN-β) in recipient cells. At the same time, caspase-1 inhibitors prevent the cleavage of GSDMD, mtRNA release, and secondary inflammatory response. A549 cells infected with VSV or pretreated with DMF and then infected with VSV, cytoplasmic RNA was isolated and transfected into fresh A549 cells, cytosolic RNA extracted from A549 cells infected with VSV alone and transfected into fresh A549 cells, induced significantly high IFN-β and IL-6 expression, compared to DMF pretreated, followed by VSV infection, cytoplasmic RNA transfected into fresh A549 cells (**Supplementary Figures S4A, B**). Similarly, in PM cells, cytosolic RNA transfected from VSV-infected cells alone into fresh PM cells significantly induced high IFN-β and IL-6 expression, in contrast to PM cells pretreated with DMF, followed by VSV infection, cytoplasmic RNA transfected into fresh PM cells (**Supplementary Figures S4C, D**). These experiments extend the relevance of mtRNA-triggered inflammation to viral infection and implicate GSDME as a parallel mediator of this response. PM cells from wild type (WT) and VISA Knockout (VISA^-/-^) mice primed with LPS, treated with Ng, cytoplasmic RNA was extracted and transfected into fresh WT and VISA^-/-^ PM cells, qPCR analysis revealed significantly high expression of IFN-β and IL-6 expression from WT PM cells treated with LPS + Ng, cytoplasmic RNA transfected into fresh WT PM cells, compared to VISA^-/-^ PM cells treated with LPS + Ng, cytoplasmic RNA transfected into fresh VISA^-/-^ PM cells (**Figures 4E, F**). These results confirm that GSDMD N-terminal mediated mitochondrial damage and mtRNA release in the cytoplasm activate the VISA pathway and induce a secondary inflammatory response. Similarly, cytoplasmic RNA from VSV-infected WT PM cells transfected into fresh WT PM cells induced significantly higher IFN-β and IL-6 expression compared to cytoplasmic RNA from VSV-infected VISA^-/-^ PM cells transfected into fresh VISA^-/-^ cells (**Supplementary Figures S4E, F**). These findings also confirmed that GSDME N-terminal mediated mitochondrial dysfunction and mtRNA release in the cytoplasm via activation of the VISA pathway induce a secondary inflammatory response. To extend these findings *in vivo*, six WT mice (three peritoneal injected with 10 mg/kg LPS and three untreated) and three VISA^-/-^ mice peritoneal injected with 10 mg/kg LPS were analyzed. Western blot analysis of lung tissues showed no GSDMD cleavage or pTBK1 expression in untreated WT mice. In contrast, LPS-injected WT mice exhibited both GSDMD cleavage and pTBK1 expression, whereas LPS-injected VISA^-/-^ mice displayed GSDMD cleavage but no pTBK1 expression. These results confirm that the VISA pathway is required for pTBK1 activation downstream of GSDMD cleavage and mtRNA release (**Figure 4G**). This *in vivo* validation highlights the physiological significance of the mtRNA-VISA pathway in systemic inflammation. Further analysis revealed that mtRNA is secreted into the extracellular space. THP-1 cells treated with LPS + Ng released significantly higher levels of mtRNA (ND5, ND6, and CYTB) into the culture media compared to VX-765-pretreated cells, supporting the role of GSDMD-N in inducing pore formation in mitochondrial and cytoplasmic membranes (**Figure 4H**). Similarly, PM cells treated with LPS + Ng released elevated mtRNA (ND1, COX1, and CYTB) into the extracellular media compared to VX-765-pretreated cells, further corroborating GSDMD-N’s role in facilitating mtRNA release (**Figure 4I**). In A549 cells infected with VSV, mtRNA (ND5, ND6, and CYTB) levels in the extracellular space were significantly higher compared to DMF-pretreated cells, indicating that DMF inhibits mtRNA release into the extracellular space by blocking GSDME activation (**Supplementary Figure S4G**). Consistent findings in PM cells revealed increased extracellular mtRNA (ND1, COX1, and CYTB) in VSV-infected PM cells compared to DMF-pretreated cells, confirming that GSDME cleavage drives mtRNA release into the extracellular environment (**Supplementary Figure S4H**). Collectively, these findings establish GSDMD and GSDME as pivotal mediators of mitochondrial dysfunction, mtRNA release, and inflammatory responses. GSDMD and GSDME cleavage led to mitochondrial and cytoplasmic membrane permeabilization, enabling mtRNA release into the cytoplasm and extracellular space. This mtRNA activates the VISA pathway in the cytoplasm to drive the upregulation of IFN-β and IL-6.

### 3.5 The significance of mtRNA and VISA pathway in mediating lung inflammation and immune responses

**Figure 5:**
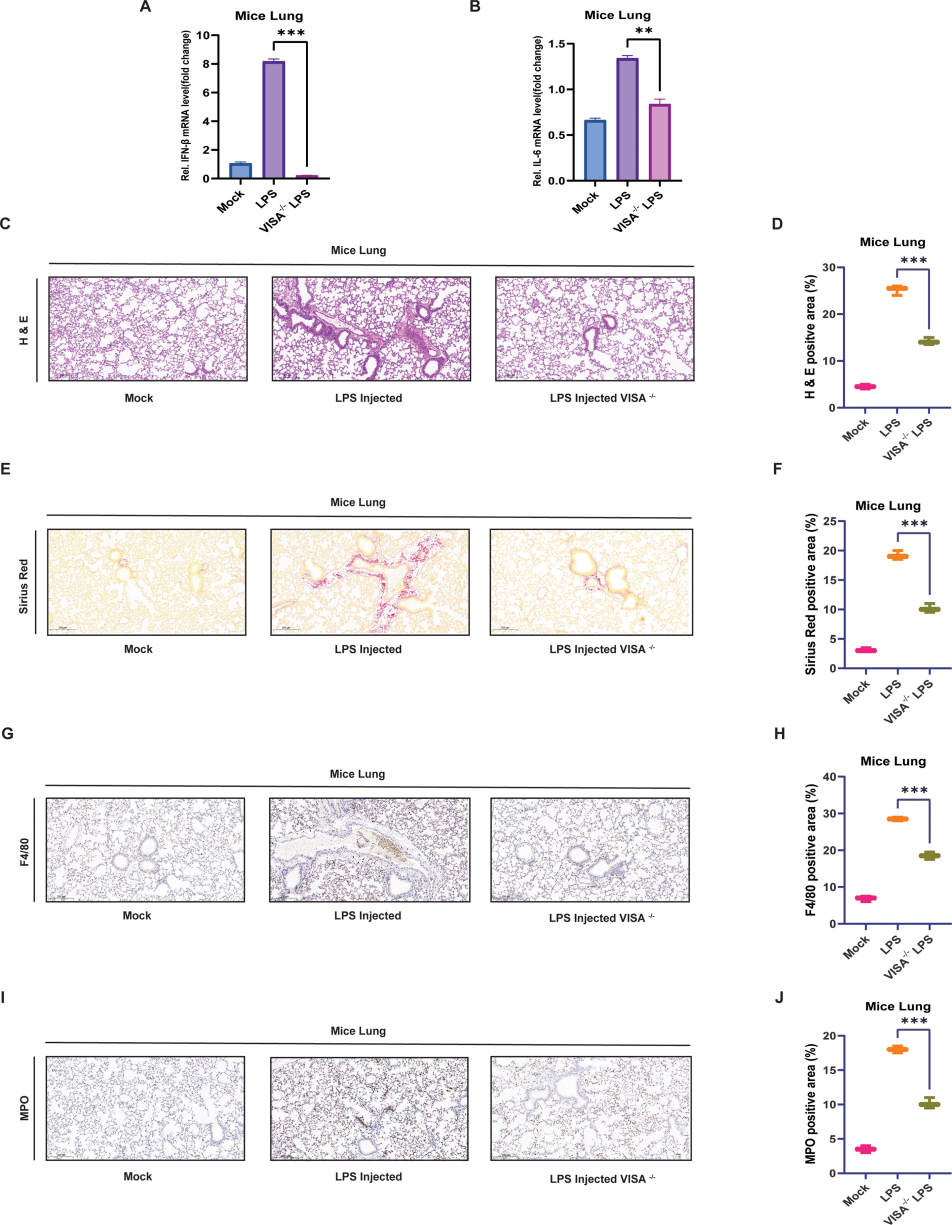
The role of mtRNA and the VISA pathway in lung inflammation, fibrosis, and immune cell recruitment. **(A, B)** Six WT mice (three peritoneal injected with 10 mg/kg LPS and three untreated) and three VISA^-/-^ mice peritoneal injected with 10 mg/kg LPS were analyzed. After 12 hours of incubation, lung tissue qPCR analysis showed significantly higher IFN-β **(A)** and IL-6 **(B)** expression in WT compared to VISA^-/-^ mice. Data are presented as mean ± SEM (***p < 0.001, **p < 0.01). Histopathological and immunohistochemical analysis of mice lung tissue. **(C, D)** Hematoxylin and Eosin (H&E) staining of lung tissues revealed that peritoneal LPS-injected WT mice displayed pronounced inflammation, including altered cellular morphology, nuclear irregularities, eosinophilia, hemorrhage, and increased alveolar spaces, indicative of pulmonary edema. In contrast, peritoneal LPS-injected VISA^-/-^ mice showed reduced inflammation. **(E, F)** Sirius Red staining of mice lung tissue revealed marked collagen deposition and pulmonary fibrosis in LPS-injected WT mice, indicative of tissue remodeling. In contrast, peritoneal LPS-injected VISA^-/-^ mice exhibited significantly reduced fibrosis. **(G, H)** Mice lung tissues were collected and subjected to immunohistochemistry (IHC) F4/80 staining to evaluate macrophage infiltration. The staining revealed extensive F4/80-positive staining in LPS-injected WT mice, indicating significant macrophage recruitment, whereas VISA^−^/^−^ mice exhibited markedly reduced macrophage infiltration. **(I, J)** Mice lung tissues were collected and analyzed using Myeloperoxidase (MPO) staining for neutrophil infiltration, revealing extensive MPO-positive staining in LPS-injected WT mice, signifying robust neutrophil recruitment. In contrast, VISA^−^/^−^ mice exhibited reduced MPO-positive staining, all experiments were repeated three times, and representative experiments are shown. Scale bar: 200 μm. Magnification: 20×. Data are representative of *n* = 3 animals per group. A total of n = 9 mice were used, including n = 6 WT mice (n = 3 untreated and n = 3 peritoneal injected with 10 mg/kg LPS) and n = 3 VISA^-/-^ mice peritoneal injected with 10 mg/kg LPS. Statistical significance was assessed using the unpaired t-test, data presented in percentage, comparing WT injected with LPS and VISA^-/-^ injected with LPS; significant differences are indicated as ***p < 0.001.

Six wild-type (WT) mice (three peritoneal injected with 10 mg/kg LPS and three untreated) and three VISA^-/-^ mice peritoneal injected with 10 mg/kg LPS were analyzed to assess inflammatory responses. After 12 hours, lung tissues were collected for qPCR and histological analysis. LPS-injected WT mice exhibited significantly higher expression of IFN-β and IL-6 compared to VISA^-/-^ mice. In contrast, untreated WT mice showed no detectable expression of these cytokines, confirming the critical role of the VISA pathway in upregulating IFN-β and IL-6 during LPS-induced inflammation (**Figures 5A, B**). To further examine the effect of LPS on lung tissue morphology, we performed H&E (Hematoxylin and Eosin) staining of mice lung tissue, showing that LPS-injected WT mice exhibited intense inflammation, including changes in cellular morphology, eosinophilia, nuclear irregularities, increased alveolar spaces, and hemorrhage, revealing pulmonary edema. In contrast, lung tissue from VISA^-/-^ mice showed reduced inflammation, further emphasizing the role of the VISA pathway in response to mtRNA release, mediating downstream inflammatory signaling (**Figures 5C, D**). In addition to inflammatory damage, Sirius Red staining of mice lung tissue revealed elevated collagen deposition and pulmonary fibrosis in WT mice injected with LPS, indicative of tissue repair. VISA^-/-^ mice demonstrated significantly reduced fibrosis. These results emphasize the VISA pathway’s important role in activating proinflammatory cytokines and type 1 interferon during inflammation, driving fibrotic responses (**Figures 5E, F**). to evaluate immune cell recruitment, F4/80 staining to analyze macrophage infiltration observed extensive F4/80 positive staining in WT mice lung tissue injected with LPS, indicative of intensive macrophage infiltration, whereas VISA^-/-^ mice injected with LPS revealed significantly reduced macrophage infiltration; these results emphasize the VISA pathway’s role in macrophage recruitment during LPS induced inflammation (**Figures 5G, H**). Similarly, MPO (myeloperoxidase) staining to analyze neutrophil infiltration revealed extensive MPO-positive staining in WT mice lung tissue injected with LPS, indicative of significant neutrophil recruitment. In contrast, lung tissue of VISA^-/-^ mice injected with LPS showed reduced MPO-positive staining, further affirming the VISA pathway’s essential role in mediating neutrophil infiltration during LPS-induced inflammatory responses (**Figures 5I, J**). Collectively, these findings highlight the pivotal role of mtRNA release into the cytoplasm, which activates the VISA pathway, triggering inflammation, fibrosis, and the recruitment of macrophages and neutrophils within the lung tissue of mice.

**Figure 6:**
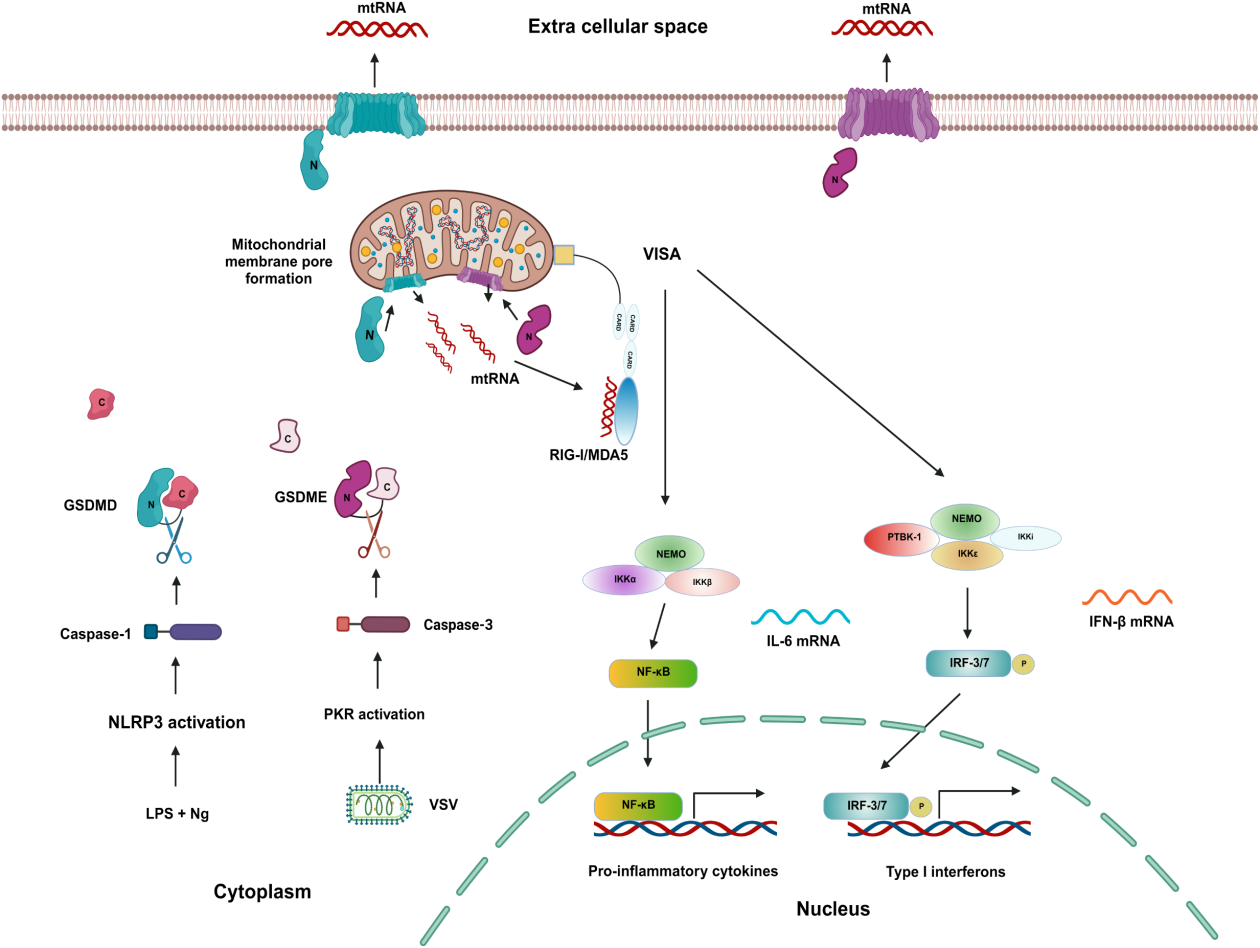
Gasdermin D and E mediated mtRNA release and VISA pathway activation induce secondary inflammatory response. Cells treated with LPS and Ng trigger the activation of the NLRP3 pathway, leading to the recruitment of caspase-1, which in turn cleaves GSDMD (31). In contrast, cells infected with VSV activate the PKR pathway and caspase-3, resulting in the cleavage of GSDME (19, 32). Our findings indicate that the N-terminal fragments of both GSDMD and GSDME facilitate mitochondrial membrane permeabilization and the release of mtRNA into the cytoplasm. This mtRNA is subsequently recognized by the double-stranded RNA sensors RIG-I and MDA5, prompting their activation (37). Activated RIG-I interacts with VISA (MAVS) on the mitochondrial membrane (38), initiating a signaling cascade that involves various kinases, including TBK-1, IKKϵ, IKKα, and IKKβ (39, 40). This cascade culminates in the phosphorylation and activation of key transcription factors, notably IRF-3/7 and NF-κB. These activated transcription factors then translocate to the nucleus to drive the expression of type I interferons (IFNs) and an array of pro-inflammatory cytokines (41, 42). Furthermore, the N-terminal fragments of Gasdermin D and E contribute to the formation of pores in the plasma membrane, facilitating the release of mtRNA into the extracellular space due to the permeabilization induced by GSDMD and GSDME.

## 4 Discussion

Mitochondrial dysfunction has emerged as a significant contributor to innate immune activation and inflammatory signaling pathways. Recent studies indicate that mitochondrial integrity plays a crucial role in immune responses, particularly through the release of mitochondrial nucleic acids into the cytoplasm, which triggers innate immune receptors and inflammasome complexes (43, 44). The gasdermin family proteins, particularly GSDMD and GSDME, have been identified as central effectors that mediate inflammatory cell death mechanisms, including pyroptosis, by creating pores in cellular membranes (45–47). However, the precise role of gasdermin-mediated mitochondrial dysfunction, mtRNA release, and its contribution to downstream inflammatory signaling remains to be fully elucidated.

Our findings expand upon this knowledge by indicating that the activation and cleavage of GSDMD through LPS + Ng and poly(dA-dT) treatment, and GSDME activation induced by VSV infection, significantly upregulates the expression of IFN-β and IL-6 in both human and mouse cells. Notably, treatment with the caspase-1 inhibitor VX-765 and the Gasdermin D and E inhibitor DMF markedly reduced these cytokines. VX-765 specifically inhibits caspase-1 activation upstream of GSDMD, while DMF directly inhibits both GSDMD and GSDME. These findings underscore the critical roles of GSDMD and GSDME as mediators of IFN-β and IL-6 production. Their inhibition attenuates this inflammatory response across human and mouse models. In contrast, previous studies have shown that the activation of caspase-11 and GSDMD in mice endothelial cells by LPS significantly upregulates IFN-β expression. However, Huang et al. reported that deletion of caspase-11 (using Casp11^fl/fl^ mice crossed with Cdh5-CreERT2 mice) or the use of GSDMD knockout (GSDMD^-/-^) mice led to a marked reduction in IFN-β levels following LPS treatment. Similar to our findings, their study also demonstrated that GSDMD cleavage induces mitochondrial dysfunction and the escape of mtDNA into the cytoplasm, where it activates the cGAS-STING pathway (21).

In line with these observations, our study showed that caspase-1 activation via LPS + Ng or LPS + poly(dA-dT) treatment, as well as the activation of GSDME by VSV infection, results in mtRNA release into the cytoplasm of both human and mouse cells. This finding aligns with Huang et al., who showed that LPS activates caspase-11 and GSDMD, leading to mtDNA release into the cytoplasm, highlighting the importance of caspase activation in mitochondrial dysfunction. Furthermore, we revealed that caspase-1 inhibition or GSDMD and GSDME inhibition significantly reduces mtRNA release into the cytoplasm. This is consistent with the observation that caspase-11^-/-^ and GSDMD^-/-^ cells exhibited low mtDNA levels, emphasizing the critical role of GSDMD and GSDME in mediating mtRNA-dependent inflammatory signaling (21). In contrast, Dhir A. et al. focused on patients with biallelic hypomorphic mutations in the PNPT1 gene, showing a different mechanism where lower PNPase levels led to mtRNA accumulation and release through Bax and Bak-mediated pathways (36). While both studies highlight the role of mitochondrial DNA or RNA in inflammation, our work explicitly emphasizes GSDMD and GSDME cleavage as key mediators. In contrast, Dhir A. et al. explore genetic mutations affecting mtRNA dynamics.

Our study further indicated that the LPS + Ng treatment activates caspase-1, and downstream cleavage of GSDMD leads to mtRNA release into the cytoplasm, which induces the IFN-β and IL-6 expression. Cells pretreated with IMT1 exhibited a marked reduction not only in mtRNA release into the cytoplasm but also reduced IFN-β and IL-6 expression, highlighting the vital role of mtRNA in driving the inflammatory response and the potential therapeutic value of IMT1 to mitigate this pathway. A similar study conducted by Hooftman et al. demonstrated that inhibition of the tricarboxylic acid cycle enzyme fumarate hydratase (FH) by the FHIN1 inhibitor strongly enhanced IFN-β production via mtRNA release-mediated, RNA sensor-dependent pathways, involving TLR7, RIG-I, and MDA5 activation. Notably, pretreated BMDMs with IMT1 had less mtRNA release in the cytoplasm and exhibited reduced IFN-β expression, paralleling our findings but highlighting different upstream signaling mechanisms involving FH inhibition (48).

Our findings showed that GSDMD cleavage occurs after stimulation with LPS + Ng, whereas GSDME is cleaved after infection with VSV in human and mouse cells. Western blotting of mitochondrial and cytoplasmic fractions confirmed that the full-length and N-terminal fragments of GSDMD and GSDME localize to the mitochondria. Additionally, JC-1 staining, viewed under inverted microscopy and flow cytometry, indicated extensive mitochondrial damage after LPS + Ng or VSV treatment. These findings suggest that N-terminal fragments of GSDMD and GSDME are accountable for mitochondrial membrane pore formation and, thus, for mitochondrial dysfunction. Consistently, Huang et al. illustrated that GSDMD activation induced by LPS treatment results in mitochondrial dysfunction, and Western blotting revealed the translocation of GSDMD-N-terminal fragments to mitochondria (21). Similarly, Yu et al. described that GSDMD cleavage induced by LPS + Ng causes mitochondrial dysfunction and that the GSDMD-N-terminal fragment is translocated into the mitochondria, where it promotes the formation of membrane pores, which consequently leads to mitochondrial injury (49). Furthermore, Rogers et al. demonstrated that caspase-3 cleavage of GSDME releases the GSDME-N terminal domain, which permeabilizes the mitochondrial membrane, causing the release of cytochrome c and activation of the apoptosome (22). These studies align with our findings that both GSDMD and GSDME contribute to mitochondrial dysfunction, reinforcing the critical role of these proteins in mediating mitochondrial damage through pore formation.

Our findings determined that the induction of IFN-β and IL-6 upregulation is primarily due to the presence of mtRNA in the cytoplasm. Specifically, both LPS + Ng-treated and VSV-infected cells released mtRNA into the cytoplasm. Transfecting cytoplasmic RNA from these cells into new cells further confirmed its role in upregulating IFN-β and IL-6 expression. In additional experiments with LPS + Ng-treated and VSV-infected VISA^-/-^ mouse cells, transfection of their cytoplasmic RNA into new VISA^-/-^ cells completely inhibited IFN-β and IL-6 expression, highlighting that mtRNA controls these expressions through the VISA pathway. These findings align with findings by Dhir et al., who identified RIG-I and MDA5 as primary mitochondrial double-stranded RNA (mt-dsRNA) sensors that signal via MAVS (VISA) to induce type I interferon responses (36). Similarly, Hooftman et al. demonstrated that mtRNA activates RIG-I and MDA5, leading to downstream inflammatory responses through MAVS (VISA) (48). However, the mechanism of mtRNA release into the cytoplasm described in their study differs from ours. We focused on the specific pathways involved in LPS + Ng and VSV treatments that led to GSDMD and GSDME cleavage, resulting in mitochondrial damage and mtRNA release. Thus, while both studies highlight the importance of mtRNA in activating the VISA pathway, the underlying mechanisms of its release into the cytoplasm vary, emphasizing the complexity of mtRNA-mediated inflammatory responses.

Our findings demonstrated that GSDMD and GSDME cleavage mediates the formation of mitochondrial pores, leading to the release of mtRNA into the cytoplasm and, notably, into the extracellular space. This highlights a dual mechanism by which gasdermin-mediated pore formation facilitates mtRNA release, potentially amplifying intracellular and extracellular inflammatory signaling. A supportive study by Kim et al. revealed that mt-dsRNA expression and its cytoplasmic escape are enhanced in chondrocytes under osteoarthritis-inducing conditions. They found that when mt-dsRNA is released into the extracellular space, it activates TLR3 present in the plasma membrane, further extending the inflammatory response (34). While both studies identify the release of mtRNA into the extracellular space, the underlying mechanisms differ: our research focuses on GSDMD and GSDME-mediated pore formation, whereas Kim et al. emphasize the context of osteoarthritis. Additionally, Kim et al. highlight the specific activation of TLR3 by mt-dsRNA, a critical aspect of their findings. This distinction underscores the complexity of mtRNA-mediated signaling and its implications in various inflammatory contexts.

Our research illustrates that WT and VISA^-/-^ mice peritoneally administered LPS exhibit distinct inflammatory responses. Western blot and qPCR analysis of WT mouse lung tissue showed GSDMD activation and robust type I interferon expression. In VISA^-/-^ mice, we observed GSDMD activation but no type I interferon expression, highlighting the significance of the VISA pathway for interferon induction. Additionally, histological examinations through H&E staining, Sirius Red staining, and immunostaining for F4/80 and MPO revealed decreased inflammation, collagen deposition, macrophage recruitment, and neutrophil infiltration in VISA^-/-^ mice compared to WT mice. These results confirm the pivotal role of the VISA pathway in regulating inflammation, macrophage recruitment, and neutrophil infiltration during LPS-induced immune stimulation. A related study by Xian et al. demonstrated that WT mice and mitochondrially targeted OGG1 (8-oxoG DNA glycosylase 1) transgenic (mt-Ogg1^Tg^) mice showed different inflammatory responses following the LPS challenge. Histopathological analysis, including H&E staining, Sirius Red staining, and F4/80 and MPO immunostaining, indicated increased inflammation, collagen deposition, macrophage recruitment, and neutrophilic infiltration in the lungs of WT mice compared to mt-Ogg1^Tg^ mice (50). While both studies observe differences in inflammatory responses between WT mice and genetically modified counterparts, our research explicitly emphasizes the role of the VISA pathway in type I interferon induction and its impact on inflammation and immune cell recruitment. In contrast, the study by Xian et al. focuses on the role of mitochondrial OGG1 in modulating inflammation, illustrating different mechanisms underlying the inflammatory responses in these contexts.

Recent studies underscore how defects in mitochondrial quality control, such as PINK1 insufficiency, can sensitize gastric adenocarcinoma cells to therapies targeting mitochondrial dynamics, linking mitochondrial distress to impaired immune responses (51). Additionally, emerging evidence highlights that tumor-associated macrophages and dendritic cells undergo significant immunometabolic reprogramming through mitochondrial signaling, influencing cancer progression and anti-tumor immunity (52). By integrating these insights, our findings not only highlight a novel innate immune pathway but also demonstrate its broader relevance across cancer biology and therapeutic innovation, positioning our work within the larger framework of cancer immunometabolism and mitochondrial signaling.

Recent studies have shown that mutations in the mitochondrial protein Cytochrome B can lead to serious health issues, such as organ malfunction and impaired immune function in livestock (53). However, the VISA pathway, activated by double-stranded RNA (dsRNA), recognizes viral RNA (54), thereby reinforcing the immune response regardless of the mtRNA’s genetic status.

Taken together, our findings underscore the central role of GSDMD and GSDME-mediated mitochondrial dysfunction in driving mtRNA release and subsequent inflammatory signaling via the VISA pathway (**Figure 6**). These results highlight gasdermins as potential targets for inflammatory diseases. Further studies should explore the precise molecular triggers, upstream regulators, and broader relevance across different inflammatory contexts to fully understand the role of gasdermin-dependent mitochondrial signaling pathways.

## 5 Conclusion

Our study demonstrates that during microbial infection, GSDMD and GSDME cleavage into their N-terminal fragments by caspase-1 and caspase-3 activation leads to mitochondrial membrane pore formation, subsequently leads to mitochondrial dysfunction and mtRNA release into the cytoplasm, in the cytoplasm self-mtRNA recognized as DAMP by primary RNA sensor VISA and upon activation induces downstream expression of inflammatory cytokines, leads to lungs tissue inflammation. These findings suggest that targeting GSDMD and GSDME by gasdermin inhibitor DMF has the potential to be an effective therapeutic approach for mitigating the harmful effects of inflammatory diseases.

While our studies offer compelling evidence for GSDMD, GSDME, and VISA pathway involvement in mtRNA-driven inflammation, further studies are required to define the exact molecular mechanisms underlying mtRNA-VISA interactions. Moreover, determining the therapeutic efficacy of VX-765 and DMF *in vivo* using disease-specific models can potentially set the stage for translational opportunities.

## Data Availability

The original contributions presented in this study are included in the article and **Supplementary Material**. Further inquiries can be directed to the corresponding author.
