# Supplementary material for: The role of gasdermin-mediated mitochondrial RNA release in amplifying secondary immune response during microbial infection
Source: Front Immunol. 2026 Jan 2;16:1668763. doi: 10.3389/fimmu.2025.1668763 (PMC12808364; doi:10.3389/fimmu.2025.1668763)
Supplement: Supplementary file 1 [file DataSheet1.pdf]

Supplementary Data

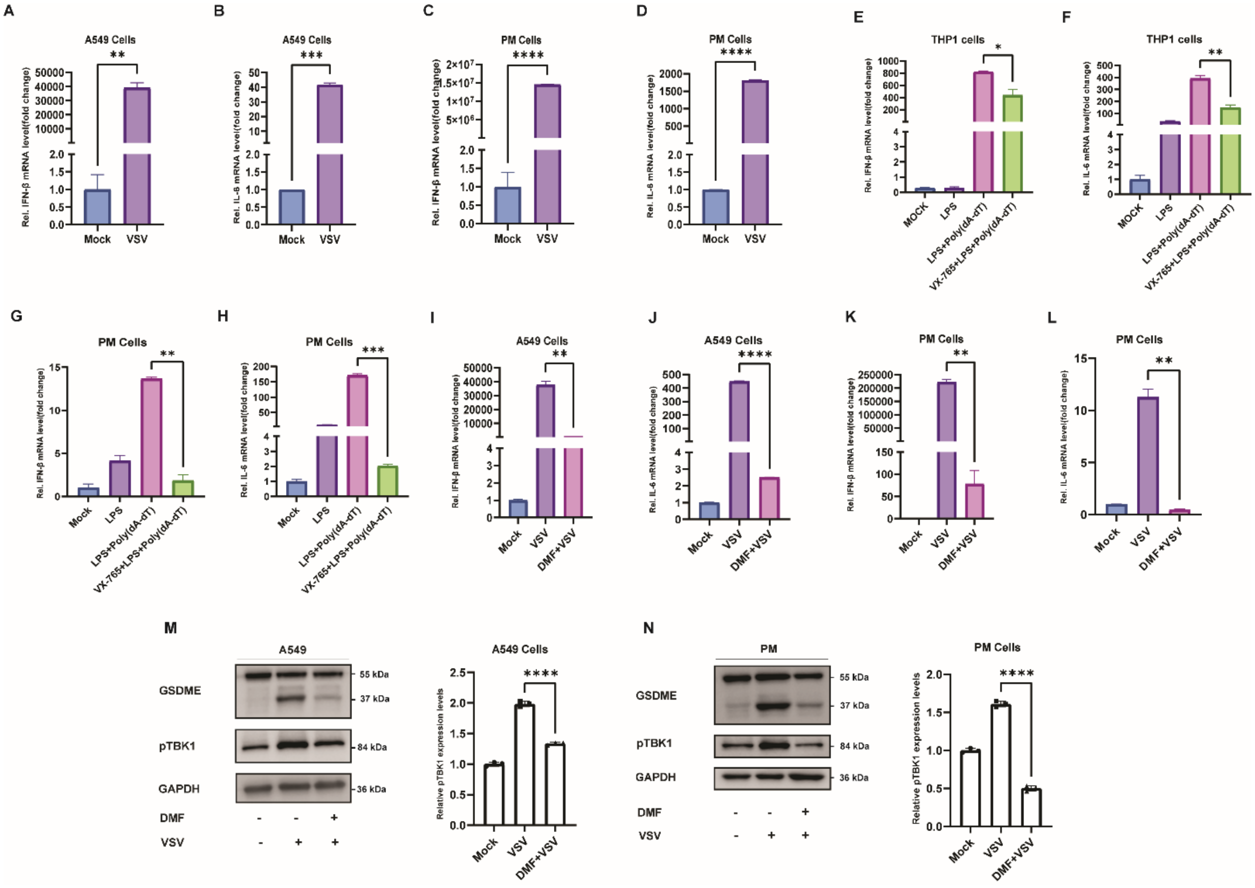

Figure-S1 Influence of Caspase-1 and GSDME Inhibition on Cytokine Expression Following Synthetic DNA and VSV Treatment

(A and B) A549 cells uninfected or infected with 20 µL, MOI = 1 VSV for 12 hours, qPCR analysis demonstrating elevated expression of IFN-β (Fig. S1A) and IL-6 (Fig. S1B), Statistical significance was determined by unpaired t-test. Data represent mean ± SEM (** $p < 0.01$, *** $p < 0.001$).

(C and D) PM cells uninfected or infected with 20 µL, MOI = 1 VSV for 12 hours, qPCR analysis demonstrating elevated expression of IFN-β (Fig. S1C) and IL-6 (Fig. S1D), Statistical significance was determined by unpaired t-test (**** $p < 0.0001$, **** $p < 0.0001$).

(E and F) THP1 cells primed with 100 ng/ml LPS alone for 2 hours, cells primed with 100 ng/ml LPS for 2 hours, then transfected with 2 µg/ml poly(deoxyadenylic-deoxythymidylic) acid (poly(dA-dT)) for 4 hours, and cells pretreated with 20 µM VX-765 for 2 hours, primed with LPS, then transfected with 2 µg/ml poly(dA-dT). qPCR analysis showing decreased IFN-β (Fig. S1E)

and IL-6 (Fig. S1F) expression in VX-765 pretreated THP-1 cells. Data represent mean ± SEM (*$p < 0.05$, **$p < 0.01$).

(G and H) PM cells primed with 100 ng/ml LPS alone for 2 hours, cells primed with 100 ng/ml LPS for 2 hours, then transfected with 2 μg/ml poly(dA-dT) for 4 hours, and cells pretreated with 20 μM VX-765 for 2 hours, primed with LPS, then transfected with 2 μg/ml poly(dA-dT). qPCR analysis showing decreased IFN-β (Fig S1G) and IL-6 (Fig S1H) expression in VX-765 pretreated PM cells. Data represent mean ± SEM (**$p < 0.01$, ***$p < 0.001$).

(I and J) A549 cells treated with 20 μL, MOI = 1 VSV alone, and cells pretreated with 100 μM/ml DMF for 4 hours and then treated with 20 μL, MOI = 1 VSV after 12 hours of incubation, qPCR analysis showing decreased IFN-β (Fig. S1I) and IL-6 (Fig. S1J) expression in DMF pretreated A549 cells. Data represent mean ± SEM (**$p < 0.01$, ****$p < 0.0001$).

(K and L) PM cells infected with 20 μL, MOI = 1 VSV alone and cells pretreated with 100 μM/ml DMF for 2 hours and then infected with 20 μL, MOI = 1 VSV after 12 hours of incubation, qPCR analysis showed decreased IFN-β (Fig. S1K) and IL-6 (Fig. S1L) expression in DMF-pretreated PM cells. Data represent mean ± SEM (**$p < 0.01$, **$p < 0.01$).

(M and N) Western blot analysis showed that VSV infection alone induced GSDME cleavage and elevated TBK1 phosphorylation in A549 and PM cells. However, prior to VSV infection, DMF pretreatment significantly inhibited GSDME cleavage and reduced TBK1 phosphorylation, pTBK1 protein expression levels were quantified using ImageJ; an unpaired t-test determined statistical significance. Data represent ± SEM (****$p < 0.0001$, ****$p < 0.0001$).

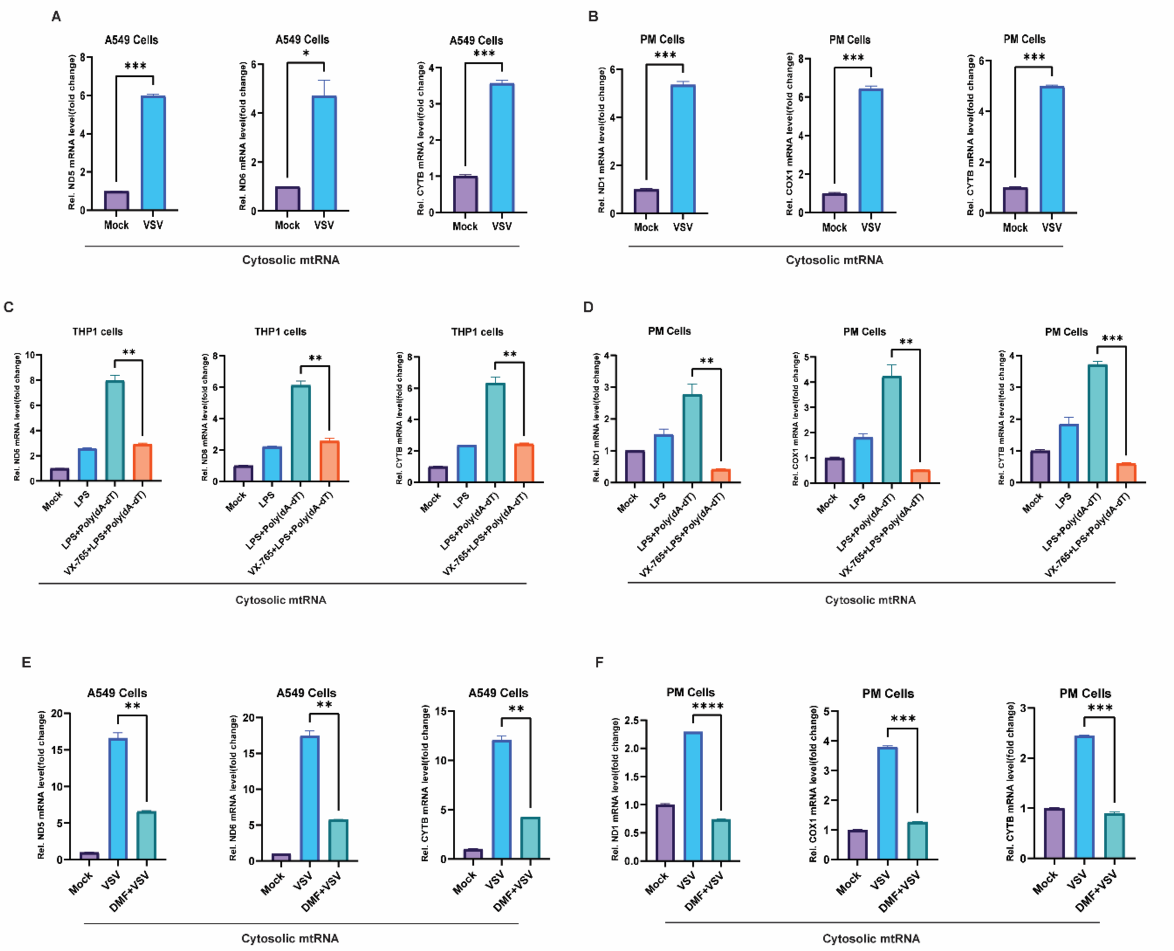

Figure S2 Caspase-1 and GSDME: Mitochondrial RNA Release Following Treatment with Synthetic Oligonucleotide and VSV

(A) In A549 cells infected with VSV and non-infected cells, cytoplasmic RNA was extracted, and qPCR analysis showed increased mtRNA (ND5, ND6, and CYTB) expression in VSV-infected A549 cells. Data represent mean ± SEM (***$p < 0.001$, *$p < 0.05$, ***$p < 0.001$).

(B) PM cells infected with VSV and non-infected cells, cytoplasmic RNA extracted, qPCR analysis showing increased mtRNA (ND1, COX1, and CYTB) expression in VSV PM cells. Data represent mean ± SEM (***$p < 0.001$, ***$p < 0.001$, ***$p < 0.001$).

(C) THP1 cells primed with LPS only, cells treated with LPS + poly(dA-dT), and cells pretreated with VX-765 then treated with LPS + Poly(dA-dT), cytoplasmic RNA extracted, qPCR analysis showing a decrease in mtRNA (ND5, ND6, and CYTB) expression in VX-765 pretreated THP1 cells. Data represent mean ± SEM (**$p < 0.01$, **$p < 0.01$, **$p < 0.01$).

(D) PM cells Primed LPS only, cells treated with LPS + poly(dA-dT), and cells pretreated with VX-765 then treated with LPS + Poly(dA-dT), cytoplasmic RNA extracted, qPCR analysis showing a decrease mtRNA (ND1, COX1, and CYTB) expression in VX-765 pretreated PM cells. Data represent mean ± SEM (**$p < 0.01$, **$p < 0.01$, ***$p < 0.001$).

(E) A549 cells infected with VSV only, cells pretreated with DMF, then infected with VSV, cytoplasmic RNA extracted, qPCR analysis showing decreased mtRNA (ND5, ND6, and CYTB) expression in DMF pretreated A549 cells. Data represent mean ± SEM (**$p < 0.01$, **$p < 0.01$, **$p < 0.01$).

(F) PM cells infected with VSV only, cells pretreated with DMF then infected with VSV, cytoplasmic RNA extracted, qPCR analysis showing decreased mtRNA (ND1, COX1, and CYTB) expression in DMF pretreated PM cells. Data represent mean ± SEM (****$p < 0.0001$, ***$p < 0.001$, ***$p < 0.001$).

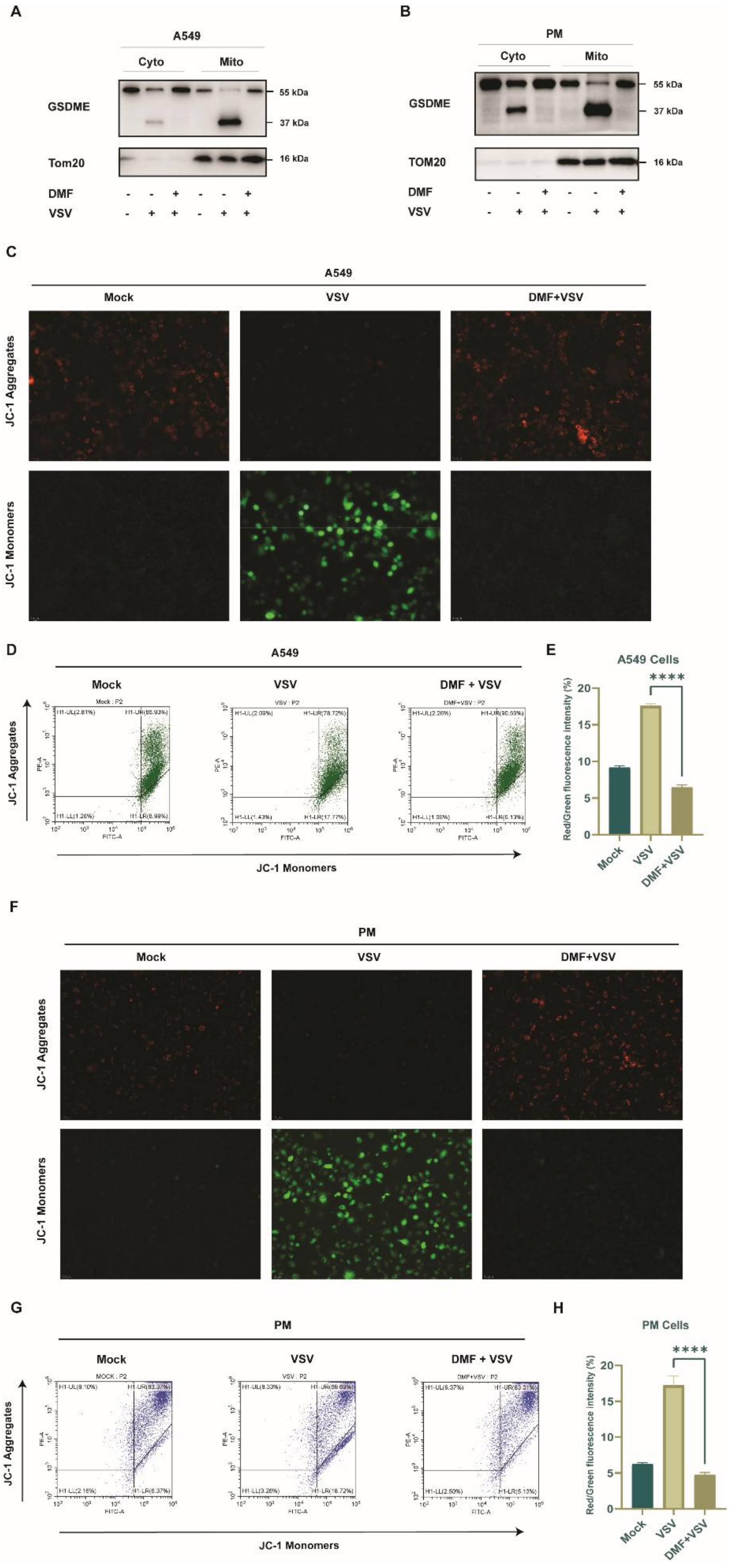

A
A549
Cyto
Mito
GSDME
55 kDa
37 kDa
Tom20
16 kDa
DMF
VSV
B
PM
Cyto
Mito
GSDME
55 kDa
37 kDa
TOM20
16 kDa
DMF
VSV
C
A549
Mock
VSV
DMF+VSV
JC-1 Aggregates
JC-1 Monomers
D
A549
Mock
VSV
DMF + VSV
JC-1 Aggregates
JC-1 Monomers
E
A549 Cells
Red/Green fluorescence intensity (%)
****
Mock
VSV
DMF+VSV
F
PM
Mock
VSV
DMF+VSV
JC-1 Aggregates
JC-1 Monomers
G
PM
Mock
VSV
DMF + VSV
JC-1 Aggregates
JC-1 Monomers
H
PM Cells
Red/Green fluorescence intensity (%)
****
Mock
VSV
DMF+VSV

Figure S3 GSDME Cleavage as Key Drivers of Mitochondrial Dysfunction:

(A) A549 cells infected or infected with VSV only, and cells pretreated with DMF then Infected with VSV, after 12 hours of incubation, underwent mitochondrial isolation and Western blot analysis showing GSDME cleavage in both cytoplasmic and mitochondrial fractions of VSV-infected A549 cells. In contrast, DMF pretreatment inhibited GSDME cleavage and mitochondrial localization.

(B) PM cells infected or infected with VSV only, and cells pretreated with DMF then Infected with VSV, after 12 hours of incubation, underwent mitochondrial isolation and Western blot analysis showing GSDME cleavage in both cytoplasmic and mitochondrial fractions of VSV-infected PM cells. In contrast, DMF pretreatment inhibited GSDME cleavage and mitochondrial localization.

(C) A549 cells infected with VSV and cells pretreated with DMF prior to VSV infection were incubated for 12 hours, followed by JC-1 staining. Inverted microscopy revealed that VSV-infected cells displayed a higher proportion of JC-1 monomers and fewer JC-1 aggregates, indicating significant mitochondrial damage. In contrast, A549 cells pretreated with DMF prior to VSV infection exhibited more JC-1 aggregates and fewer monomers.

(D and E) A549 cells infected with VSV and cells pretreated with DMF prior to VSV infection and mitochondrial damage were assessed using JC-1 staining. Flow cytometry analysis confirmed the results observed by inverted microscopy; the histogram shows the percentage.

(F) PM cells infected with VSV and cells pretreated with DMF prior to VSV infection, followed by JC-1 staining. Inverted microscopy revealed that VSV-infected cells displayed a higher proportion of JC-1 monomers and fewer JC-1 aggregates, indicating significant mitochondrial damage. In contrast, PM cells pretreated with DMF prior to VSV infection exhibited more JC-1 aggregates and fewer monomers.

(G and H) PM cells infected with VSV and cells pretreated with DMF prior to VSV infection and mitochondrial damage were assessed using JC-1 staining. Flow cytometry analysis confirmed the results observed by inverted microscopy; the histogram shows the percentage.

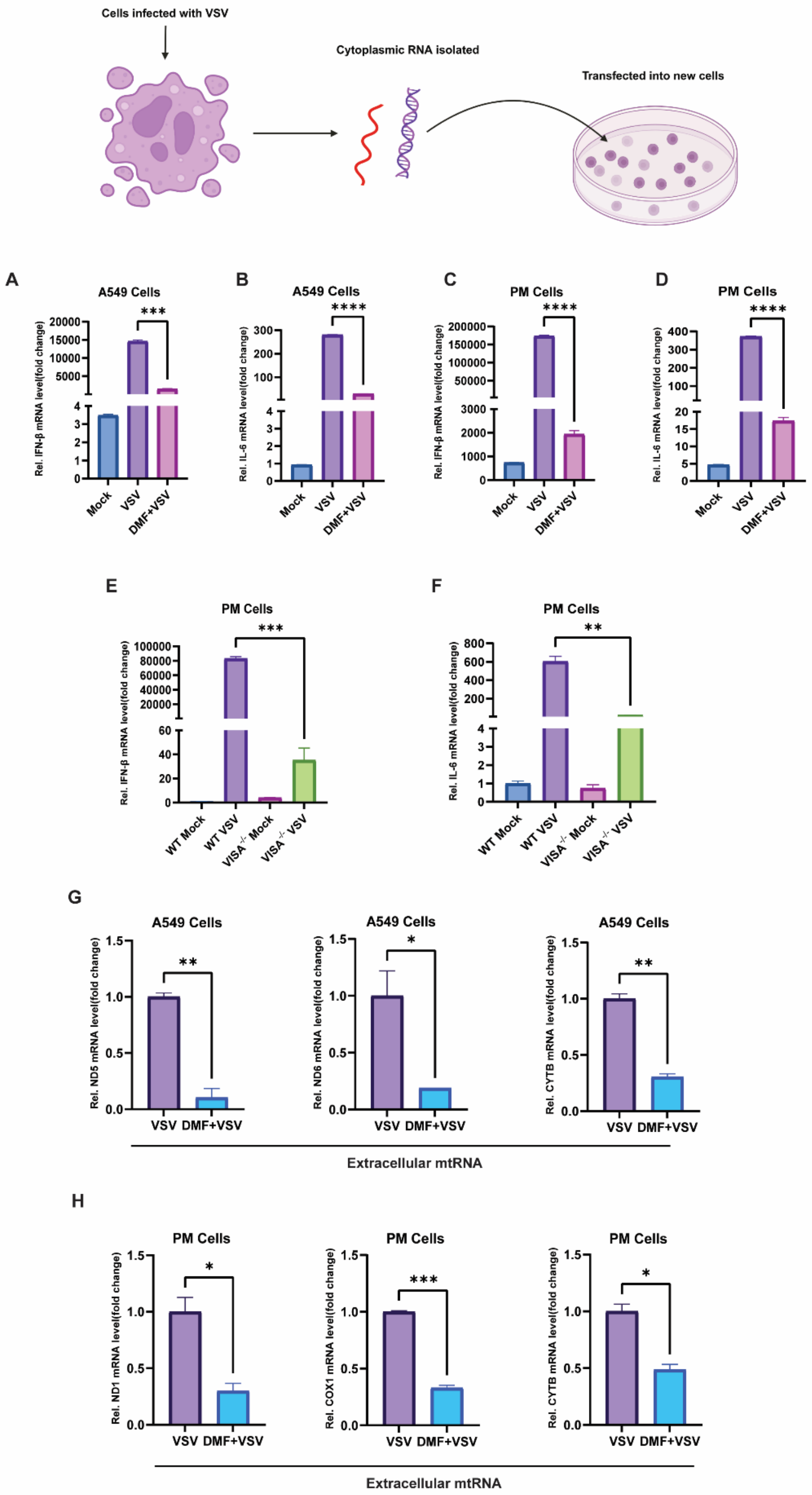

Cells infected with VSV
Cytoplasmic RNA isolated
Transfected into new cells
A
A549 Cells
***
Rel. IFN-β mRNA level(fold change)
Mock
VSV
DMF+VSV
B
A549 Cells
****
Rel. IL-6 mRNA level(fold change)
C
PM Cells
****
Rel. IFN-β mRNA level(fold change)
D
PM Cells
****
Rel. IL-6 mRNA level(fold change)
E
PM Cells
***
Rel. IFN-β mRNA level(fold change)
WT Mock
WT VSV
VISA-/- Mock
VISA-/- VSV
F
PM Cells
**
Rel. IL-6 mRNA level(fold change)
G
A549 Cells
Rel. ND5 mRNA level(fold change)
Rel. ND6 mRNA level(fold change)
Rel. CYTB mRNA level(fold change)
VSV DMF+VSV
Extracellular mtRNA
H
PM Cells
Rel. ND1 mRNA level(fold change)
Rel. COX1 mRNA level(fold change)
Rel. CYTB mRNA level(fold change)
VSV DMF+VSV
Extracellular mtRNA

Figure S4 GSDME-Mediated mtRNA Release Induces a Secondary Inflammatory Response through the VISA Pathway

(A and B) In A549 cells infected with VSV and cells pretreated with DMF prior to VSV infection, cytoplasmic RNA was extracted, and 2 μg/ml RNA was transfected into fresh cells. qPCR analysis showed significantly higher IFN-β (Fig. S3E) and IL-6 (Fig. S3F) expression in fresh A549 cells transfected from VSV infected alone compared to DMF-pretreated cells. Data are presented as mean ± SEM (***$p < 0.001$, ****$p < 0.0001$).

(C and D) In PM cells infected with VSV and cells pretreated with DMF prior to VSV infection, cytoplasmic RNA was extracted, and 2 μg/ml RNA was transfected into fresh cells. qPCR analysis showed significantly higher IFN-β (Fig. S3G) and IL-6 (Fig. S3H) expression in fresh PM cells transfected from infected with VSV alone compared to DMF-pretreated cells. Data are presented as mean ± SEM (****$p < 0.0001$, ****$p < 0.0001$).

(E and F) Wild-type (WT) PM cells and VISA knockout ($VISA^{-/-}$) PM cells infected with VSV had their cytoplasmic RNA extracted, and 2 μg/ml RNA was transfected into fresh WT PM cells and $VISA^{-/-}$ PM cells. qPCR analysis showed significantly higher IFN-β (Fig. S3I) and IL-6 (Fig. S3J) expression in fresh WT PM cells than in fresh $VISA^{-/-}$ PM cells. Data are presented as mean ± SEM (***$p < 0.001$, **$p < 0.01$).

(G) A549 cells were infected with VSV, and cells were pretreated with DMF, followed by VSV infection, and their culture media was collected after 12 hours. RNA extraction and qPCR analysis revealed higher expression of mtRNA (ND5, ND6, and CYTB) in the extracellular space of VSV-infected cells compared to DMF-pretreated cells. Data are presented as mean ± SEM (**$p < 0.01$, *$p < 0.05$, **$p < 0.01$).

(H) PM cells infected with VSV and cells were pretreated with DMF, followed by a VSV infection, and their culture media was collected. RNA extraction and qPCR analysis revealed higher expression of mtRNA (ND1, COX1, and CYTB) in the extracellular space of VSV-infected cells compared to DMF-pretreated cells. Data are presented as mean ± SEM (*$p < 0.05$, ***$p < 0.001$, *$p < 0.05$).
